# Tau Axonal Sorting and Interaction With Synaptic Plasticity Modulators Is Domain‐ and Isoform‐Dependent in Human iPSC‐Derived Neurons

**DOI:** 10.1111/acel.70215

**Published:** 2025-09-24

**Authors:** Michael Bell‐Simons, Helen Breuer, Laura Wunderlich, Hanin Chmes, Daniel Adam, Jennifer Klimek, Sarah Buchholz, Hans Zempel

**Affiliations:** ^1^ Institute of Human Genetics University Hospital Cologne Cologne Germany; ^2^ Center for Molecular Medicine Cologne (CMMC) University of Cologne Cologne Germany

**Keywords:** Alzheimer's disease, human *MAPT* knockout, proline‐rich region 2, synaptic tau, tau isoforms, tau sorting

## Abstract

Somatodendritic missorting of the axonal microtubule‐associated protein Tau is an early hallmark of Alzheimer's disease (AD) and other tauopathies. Tau missorting causes synaptic loss and neuronal dysfunction, but the mechanisms underlying both normal axonal sorting and pathological missorting remain unclear. The six human brain Tau isoforms show different axodendritic distribution, but the Tau domains governing intracellular sorting and essential interactors are unknown. Here, we aimed to identify domains or motifs of human Tau and cellular binding partners required for efficient axonal Tau sorting and to unravel isoform‐specific Tau interactors. Using human *MAPT*‐KO induced pluripotent stem cell (iPSC)‐derived glutamatergic neurons, we analyzed the sorting behavior of more than 20 truncation‐ or phosphorylation‐mutant Tau constructs and used TurboID‐based proximity labeling and proteomics to identify sorting‐ and isoform‐specific Tau interactors. We found that efficient axonal Tau sorting was independent of the N‐terminal tail, the C‐terminal repeat domains, AD‐associated phosphorylation, and the general microtubule affinity of Tau, but it requires the presence of the proline‐rich region 2 (PRR2). Our interactome data revealed peroxisomal accumulation of the Tau N‐terminal half, while axonal Tau interacted with the PP2A activator HSP110. Further, we found 0N4R‐specific interactions of Tau with regulators of presynaptic exocytosis and postsynaptic plasticity, which are partially associated with AD pathogenesis, including members of the CDC42 pathway and the RAB11 proteins, while 0N3R‐Tau bound to various cytoskeletal elements. In sum, our study i) postulates that axonal Tau sorting relies on the PRR2 domain but not on microtubule affinity and ii) unravels a potential isoform‐specific role in synaptic function and AD‐related dysfunction.

## Graphical abstract

Efficient axonal Tau sorting requires the PRR2 domain but does not depend on AT8 or KXGS phosphorylation, the MT affinity, the C‐terminal repeat domains, or the N‐terminal tail of Tau. Human 0N4R‐Tau shows isoform‐specific interaction with modulators of synaptic plasticity involved in spine function under both normal and AD conditions.

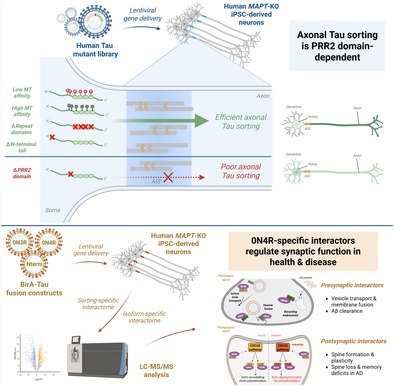

## 1 Background

The microtubule (MT)‐associated protein Tau regulates the dynamic assembly and disassembly of axonal MT filaments in neurons (Binder et al. 1985; Cleveland et al. 1977b; Kempf et al. 1996; Weingarten et al. 1975). Tau binds or dissociates from MT filaments depending on its phosphorylation state to control MT stability (Biernat et al. 1993; Drechsel et al. 1992; Lindwall and Cole 1984), thereby regulating axonal outgrowth, cargo transport, and other essential neuronal functions (Brandt et al. 1995; Dawson et al. 2010; Dixit et al. 2008; Drubin et al. 1985; Morris et al. 2015). The six Tau isoforms of the human brain (Cleveland et al. 1977a, 1977b; Goedert et al. 1989) vary in their MT affinity and are thought to have distinct roles in Tau function (Buchholz and Zempel 2024; Bullmann et al. 2009).

Tau is associated with many neurodegenerative diseases called tauopathies (Arendt et al. 2016; Spillantini et al. 1997). In Alzheimer's disease (AD), the most prevalent tauopathy, pathological Tau changes are a key disease hallmark (Arendt et al. 2016; Guo et al. 2017; Tracy and Gan 2018). So‐called Tau pre‐tangles, high levels of missorted Tau with phosphorylation patterns typical for Tau aggregates in AD brains, manifest decades before disease onset (Braak and del Tredici 2011; Braak et al. 2011). In vitro studies revealed that pathological Tau missorting has two distinct effects. Axonal function gets disturbed (Ishihara et al. 1999; Zhang et al. 2004), presumably due to MT destabilization and failed motor transport (Morris et al. 2015; Scholz and Mandelkow 2014; Tracy and Gan 2017), and abnormal dendritic Tau induces postsynaptic MT breakdown, excitotoxicity, and spine loss (Ittner and Ittner 2018; Zempel et al. 2013; Zempel and Mandelkow 2015).

Hence, prevention of Tau missorting bears potential for future therapeutic avenues, but the general principles of axonal Tau sorting, even under healthy conditions, are poorly understood. To date, several models are proposed (Zempel and Mandelkow 2019), such as anterograde transport either by free diffusion (Konzack et al. 2007), by MT‐dependent transport (Utton et al. 2002; Utton et al. 2005; Zhang et al. 2004) or yet unknown mechanisms. Axonal Tau retention by compartment‐specific modifications or interactors like annexins was reported (Chudobová and Zempel 2023; Gauthier‐Kemper et al. 2018; Zempel and Mandelkow 2019) while work with traceable Tau revealed a selective retrograde diffusion barrier within the axon initial segment (AIS) (Li et al. 2011), a key structure of neuronal polarity (Leterrier 2018; Rasband 2010). This Tau barrier depends on polymerized MTs (Li et al. 2011; Van Beuningen et al. 2015; Zempel et al. 2017), unlike the general AIS‐based protein filter, which is based on f‐actin (Kole et al. 2008; Song et al. 2009).

The Tau domains responsible for axonal Tau sorting are largely unknown. The reported relevance of MT stability (Li et al. 2011; Mercken et al. 1995; Scholz and Mandelkow 2014) hints at the MT‐binding domains of Tau, consisting of the C‐terminal repeat domains as core region and its flanking domains, especially the proline‐rich region 2 (PRR2) (Amos 2004; Gustke et al. 1994). Tau phosphorylation of the AT8 motif within PRR2 or of the KXGS motifs within the repeat domains decreases MT affinity of Tau and increases protein mobility (Biernat et al. 1993; Drechsel et al. 1992; Yoshida and Ihara 1993). Hyperphosphorylation of AT8 and KXGS residues is associated with Tau missorting and aggregation in AD (Bancher et al. 1989; Mercken et al. 1992; Zempel and Mandelkow 2014; Zempel et al. 2010). Interestingly, recombinant Tau with low MT affinity, caused by KXGS pseudophosphorylation and repeat domain depletion, was efficiently sorted in rodent primary neurons (Iwata et al. 2019; Zempel et al. 2017). In contrast, mutations in all PRR2 phosphorylation sites prevented anterograde Tau transit (Iwata et al. 2019), strengthening the idea that tight MT binding, unlike low MT affinity, impairs Tau sorting.

Notably, the six Tau isoforms show remarkable differences in sorting efficiency (Bachmann, Bell, et al. 2021; Zempel et al. 2017), and there is growing evidence for isoform‐specific roles of Tau in health and disease (Buchholz and Zempel 2024). However, data about isoform‐specific interactions in human neurons are missing (Kavanagh et al. 2022).

Besides the lack of human neuronal models, which impedes accurate Tau sorting research, previous studies on Tau sorting suffer from other experimental limitations, including the focus on unidirectional Tau transit, the use of a small subset of Tau mutants, and the expression of large Tau/reporter fusion constructs. Moreover, the most common non‐human cell models fail to resemble key aspects of human Tau physiology (Janke et al. 1999; Kavanagh et al. 2022; Xia et al. 2016).

Here, we aimed to decipher the Tau sorting process in human Tau‐depleted iPSC‐derived glutamatergic neurons. We generated a Tau library of truncated and phospho‐mutant Tau fragments and used lentiviral delivery to express them without background protein. Our findings revealed that efficient axonal Tau sorting is independent of the N‐terminal tail, the C‐terminal repeat domains, and the general MT affinity and AT8/KXGS phosphorylation state of Tau, but requires the presence of the proline‐rich region 2 (PRR2). We then used TurboID proximity labeling to compare the interactome of axonally sorted and non‐sorting Tau constructs and identified the PP2A activator HSP110 as an axon‐specific Tau interactor. Lastly, we examined the interactomes of two human Tau isoforms and found 0N4R‐specific interactions with regulators of presynaptic exocytosis and postsynaptic plasticity associated with AD pathogenesis, such as members of the CDC42 pathway and the RAB11 proteins.

In sum, our study postulates that axonal Tau sorting relies on the PRR2 domain but not on MT affinity or the C‐terminal repeat domains, and unravels a potential isoform‐specific role in synaptic function and AD‐related dysfunction. These novel insights have major implications for the targeted development of remedies for Tau pathology/missorting‐associated diseases.

## 2 Methods

### 2.1 Molecular Biology

#### 2.1.1 Generation of Lentiviral Expression Plasmids

Mutant HA‐tagged Tau and BirA‐Tau constructs were engineered with site‐directed mutagenesis, HA‐tagged BirA‐Tau constructs by linking Tau with HA‐tagged BirA (Addgene #107171) using the NEBuilder HiFi DNA Assembly Kit (NEB). All constructs were subcloned into the pJET2.1 vector, transferred to the lentiviral expression vector pUltra‐dox, and validated by Sanger sequencing. All Tau^HA^ and BirA‐Tau^HA^ constructs were cloned downstream of the cDNA sequence encoding for dTomato, coupled via a P2A peptide sequence.

### 2.2 Cell Culture

#### 2.2.1 Maintenance of Human iPSCs

WTC11 cells with a doxycycline (doxy)‐inducible *Ngn2* transgene ‘WT iPSCs’ (Miyaoka et al. 2014; Wang et al. 2017; Zhang et al. 2013), and a subclone from this line with CRISPR/Cas9‐generated biallelic *MAPT* knockout (‘*MAPT*‐KO iPSCs’) were used (Buchholz et al. 2022). Both iPSC lines were cultivated as previously described (Bell et al. 2021; Buchholz, Bell‐Simons, Cakmak, et al. 2024). Briefly, cells were cultured on GelTrex‐coated plates (Thermo Fisher) in iPS‐Brew XF (StemMACS, Miltenyi) supplemented with Anti/Anti (1×, Thermo Fisher). Cultures were passaged with Versene (ThermoFisher) when confluent, seeded in thiazovivine‐supplemented (2 μM, Axon Medchem) iPS‐Brew XF, and further cultured without thiazovivine.

#### 2.2.2 Differentiation of hiPSC‐Derived Cortical Glutamatergic Neurons

Differentiation of iPSCs into cortical glutamatergic neurons was done as previously described (Bachmann, Linde, et al. 2021; Buchholz, Bell‐Simons, Cakmak, et al. 2024) with slight adaptations (Figure S1a). Briefly, cells were seeded (1.5–2 × 10^5^ cells per cm^3^) onto GelTrex‐coated plates with doxy‐containing (2 μg/mL) medium, and after daily medium change, seeded (2.5 × 10^4^ cells/cm^3^ for immunostaining, 4–5 × 10^4^ cells/cm^3^ for protein harvest) onto culture plates coated with 50 μg/mL Poly‐D‐Lysine (PDL, Sigma Aldrich) and 20 μg/mL Cultrex 3D‐laminin (Sigma Aldrich), in doxy‐containing (2 μg/mL) medium. From week 3 on, cultures were grown in doxy‐free medium. Weekly exchange of half the medium was continuously done.

#### 2.2.3 Cultivation of HEK293T Cells

HEK293T were cultured under standard atmosphere (37°C, 5% CO_2_) in high‐glucose DMEM with GlutaMAX (Thermo Fisher) supplemented with 10% fetal bovine serum (FBS, Biochrom AG) and Anti/Anti (1X). Confluent cultures were passaged using 0.05% Trypsin/0.2% EDTA (Pan Biotech).

#### 2.2.4 Lentivirus Production

Lentiviral particles were produced as previously described (Buchholz, Bell‐Simons, Cakmak, et al. 2024) with slight modifications. In brief, HEK293T cells were seeded and grown to 60%–80% confluency (day 0). 10 μg of the lentiviral expression plasmids pUltra (Addgene #24129), pUltra‐chili (Addgene #48687), or pUltra‐dox (Addgene #58749) were mixed with the helper plasmids pMD2.G (9 μg, Addgene #12259) and psPAX2 (1 μg, Addgene #12260), and 20 μL of polyethylenimine (PEI, Thermo Fisher) in DMEM without supplements. After 20 min, the mixture was added to the cultures in 5 mL of fresh medium. After replacing the medium (day 2), virus‐containing medium was collected, centrifuged for 5 min at 400 g, filtered (0.45 μm pore size), and stored at −80°C (day 4). The virus harvesting was repeated once for each culture (day 5).

For virus titering, HEK cells were seeded onto 24‐well plates, transduced after 4–5 h with different virus dilutions, and fixed after 48 h by adding 7.4% formaldehyde. For pUltra‐dox, a rtTA‐encoding virus (Addgene # 58750) and 2 μg/mL doxy were added. After staining with NucBlue (ThermoFisher), all cultures were imaged with ZOE fluorescent cell imager (Biorad), and dTomato‐positive neurons were counted (Fiji/ImageJ V1.53t, NIH) for cultures with 5 to 30% positive neurons to avoid biased measurements. The titer was calculated as follows:
(1)

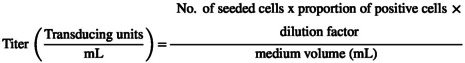




#### 2.2.5 Transduction of WT iPSC‐Neurons

WT iPSC‐neurons were transduced with pUltra/pUltra‐chili lentiviruses at different ages as previously described (Buchholz, Bell‐Simons, Cakmak, et al. 2024). Virus particles were thawed on ice, mixed with pre‐warmed and pH‐adjusted medium (50% fresh, 50% conditioned), added for 16–24 h, and replaced with maturation medium (50/50), containing 2 μg/mL doxy when cultures were younger than 3 weeks. Young neurons (week 1–3) were transduced for 3–4 days, while older neurons (week 4–10) 7–10 days before fixation.

#### 2.2.6 Tau^HA^ Construct Expression

6‐weeks‐old *MAPT*‐KO iPSC‐neurons were transduced with pUltra‐dox lentiviruses with Tau^HA^ constructs as described for WT iPSC‐neurons, with modifications. After incubation, the virus was replaced with doxycycline‐containing (0.5 μg/mL) maturation medium (50/50). For protein isolation, 4000 TU were added to one 10 cm dish (3 × 10^6^ cells). For staining, 100–200 TU were added to 24‐well plates (5 × 10^4^ cells, with coverslip). Cells were fixed or harvested 12–13 days after transduction stopped.

#### 2.2.7 BirA‐Tau^HA^ Construct Expression and Biotinylation

Transduction and expression of BirA‐Tau^HA^ was performed as described above for Tau^HA^ constructs, with modifications. Virus particles were added in biotin‐free medium with NS21 (Pan Biotech) instead of B27 and DMEM/F‐12 instead of Neurobasal. For protein isolation, 10,000 TU were added per dish, and for staining, 340 TU per well. After 12–13 days, fresh medium containing 500 μM biotin (Sigma‐Aldrich) was added, and neurons were incubated for 20 min and fixed or harvested immediately.

### 2.3 Protein Biochemistry

#### 2.3.1 Protein Isolation

The protein isolation was adapted from previous studies (Cho et al. 2020) with modifications. *MAPT*‐KO iPSC‐neurons were washed twice with cold DBPS, and pre‐lysed with cold RIPA buffer (Thermo Fisher) supplemented with 1× cOmplete (Sigma‐Aldrich) and 1 mM phenylmethylsulfonyl fluoride (PMSF, Sigma‐Aldrich) for 1.5 min. Cells were collected, homogenized by pipetting, and lysed for 20 min shaking at 4°C. The lysates were centrifuged for 10 min at 13,000 g, and stored at −80°C. Protein concentration was determined using a bichinonic acid (BCA) protein assay kit (Thermo Fisher).

#### 2.3.2 Western Blot Analysis

Proteins were diluted in 6× protein loading buffer containing (Cho et al. 2020) and boiled for 10 min at 95°C. Proteins were separated on a 10% polyacrylamide running gel (Mini‐Protean system, BioRad) and blotted to PVDF membranes (Bio‐Rad) by wet transfer (30 V, 4°C). The membrane was blocked in 5% non‐fat dry milk, incubated with the mouse anti‐HA (16B12) antibody (1:5000, #901533, BioLegend) in milk or 3% BSA, washed thoroughly, and incubated with an HRP‐coupled secondary antibody (Thermo Fisher). Detection was done with SuperSignal West Pico solutions (Thermo Fisher) using a ChemiDoc XRS+ (Bio‐Rad). For normalization, signals from HRP‐coupled anti‐GAPDH (ab9385, Abcam) and anti‐vinculin (#18799, Cell Signaling) antibodies were detected.

### 2.4 Imaging Analysis

#### 2.4.1 Fixation and Immunostaining

Neuron cultures were fixed and stained as previously described (Buchholz, Bell‐Simons, Haag, and Zempel 2024). In brief, formaldehyde (FA)‐fixed cultures were permeabilized and blocked with 5% bovine serum albumin (BSA, Sigma‐Aldrich) and 0.1% Triton X‐100 (AppliChem), incubated with the primary antibodies (rabbit anti‐Tau (K9JA), 1:1000, #A0024, DAKO, chicken anti‐MAP2, 1:2000, ab5392, Abcam, mouse anti‐HA (16B12), 1:1000, #901533, BioLegend), washed thoroughly, and incubated with the corresponding AlexaFluor coupled secondary antibody (Thermo Fisher). For biotin detection, a NeutrAvidin‐AF647 conjugate was generated as described previously (Cho et al. 2020). To ensure comparability, samples of each independent experiment were always treated with the same blocking and antibody solutions for identical times. Nuclei were stained with NucBlue (1 drop/mL, Hoechst 33342, TFS), and cultures were mounted with aqueous PolyMount (#18606, Polysciences). After 24 h of drying, microscope slides were stored at 4°C in the dark.

#### 2.4.2 Fluorescence Microscopy

Neurons in culture were imaged with ZOE (Bio‐Rad); immunostained neurons were imaged using an Axioscope 5 setup (Zeiss) (Bell et al. 2021). Exposure time and light intensity were optimized to prevent sample bleaching and saturation of fluorescent signals. Exposure and light settings were kept identical per replicate for Tau^HA^ and BirA‐Tau^HA^ detection, and exposure times were the same per replicate for dTomato and Tau^HA^ or BirA‐Tau^HA^ channels.

### 2.5 Image Analysis

Image analysis was performed using Fiji/ImageJ (V1.53t, NIH). All images were blinded using the Fiji blind analysis tool.

#### 2.5.1 Sorting Analysis of Endogenous Tau/MAP2

A somatic patch without nuclear overlap was manually set as the somatic region of interest (ROI) per neuron. The axonal ROI spanned over 5–10 μm at 75–100 μm distance to the soma, the dendritic ROI over 5–10 μm at 25–50 μm distance, the nuclear ROI comprised the NucBlue‐positive area. For all ROIs, the mean intensity of anti‐Tau, anti‐MAP2, and the reporter protein dTomato/GFP was measured (see Data S2). Background ROIs near the four ROIs were subtracted from raw ROI values. To obtain the axonal enrichment factor (AEF) of Tau and MAP2, the axon‐to‐soma ratio of Tau/MAP2 was divided by the axon‐to‐soma ratio of dTomato. Dendritic enrichment factor (DEF) and nuclear enrichment factor (NEF) were determined accordingly. Neurons that did not reach all channel‐specific detection thresholds were excluded from analysis. Exceptionally, young neurons (≤ 2 weeks) were included for axonal and nuclear enrichment analysis even with sub‐threshold signals of dendritic ROIs, due to the modest dendritic outgrowth in many young neurons. AEF, DEF, and NEF values were averaged per replicate as follows:

(2) $$\text{Mean}=\frac{\mathrm{Sum}\;\left(\text{values}>1\right)+\text{Number}\;\left(\text{values}<1\right)}{\mathrm{Sum}\;{\left(\text{Values}<1\right)}^{-1}+\text{Number}\;\left(\text{values}>1\right)}$$

#### 2.5.2 Sorting Analysis of Tau^HA^ and BirA‐Tau^HA^ Constructs

Tau^HA^ construct sorting was analyzed like endogenous Tau, with slight differences. Anti‐HA signals of three random non‐transduced neurons were subtracted from all ROIs prior to calculation. Neurons not reaching all channel‐specific detection thresholds were excluded from analysis. For BirA‐Tau^HA^ neurons, anti‐HA and NeutrAvidin‐AF647 of three random non‐transduced neurons were subtracted from all ROIs prior to calculation. Neurons not reaching all channel‐specific detection thresholds were excluded from analysis.

#### 2.5.3 Relative Protein Levels of Tau^HA^ and BirA‐Tau^HA^ Constructs

Tau^HA^ and BirA‐Tau^HA^ constructs were co‐expressed with the 2A‐coupled dTomato in equimolar ratios. For determining protein levels relative to dTomato, somatic/axonal ROIs of the anti‐HA channel were summed per replicate and divided by the somatic/axonal ROI sum of dTomato. The protein levels of each replicate were averaged by using Formula (2).

#### 2.5.4 Correlation of Tau^HA^ /BirA‐Tau^HA^ Expression Levels and the AEF

Protein levels of Tau^HA^ and BirA‐Tau^HA^ constructs were correlated to their AEF with simple linear regression analysis. Protein levels and AEFs were normalized to the replicate maximum AEF and protein level, and all neurons were grouped and analyzed together for each construct.

#### 2.5.5 AIS Development in WT iPSC‐Derived Neurons

Accumulation of ANKG and TRIM46 at the proximal axon as well as axonal MAP2 levels was determined in WT neurons at different days of differentiation. Fixed neurons were immunostained, and plot profiles were determined. To this end, a linear ROI (80 μm length, 3 pt. thickness) was used to trace the proximal axon and measure the fluorescent signals. An identical ROI next to the axon was subtracted as background. For the calculation of AIS enrichment of ANKG and TRIM46, the peak intensity (mean of all values > 70% of maximum value) was divided by the baseline intensity (mean of the most distal values, same range width as peak intensity).

#### 2.5.6 Overexpression Levels of 0N3R‐Tau^HA^

For determining the overexpression of 0N3R‐Tau^HA^ in *MAPT*‐KO neurons compared to endogenous Tau in WT neurons, absolute protein levels were determined with total Tau antibody (K9JA) using identical acquisition settings, and somatic and axonal protein levels were either measured individually or summed up.

#### 2.5.7 Subcellular Distribution of dTomato

Four‐weeks‐old human WT iPSC neurons were transduced with dTomato‐expressing lentiviruses 7 days before imaging and incubated with 5 μM of CellTrace CFSE cell tracking dye (#C34554, Thermo Fisher) for 30 min before imaging. Neurons were imaged in culture with a Leica Thunder fluorescence microscope (Leica Microsystems), and plot profiles (axons: 75 μm length, dendrites: 65 μm length, 3 pt. thickness) of the proximal axons and dendrites were determined for dTomato and CFSE.

#### 2.5.8 Okadaic Acid (OA) Treatment

Three‐weeks‐old human WT iPSC neurons were treated with OA in different concentrations for 3 h, then fixed and immunostained. Somatic levels of total Tau (K9JA) and phosphorylated Tau (AT8) were imaged with the Axioscope setup (see section above) using z‐stack acquisition by the Apotome 3 (Zeiss), and then quantified by using maximum projection intensity in ImageJ/Fiji.

#### 2.5.9 Statistical Analysis

All results of the sorting analysis for endogenous Tau, MAP2, overexpressed Tau^HA^ or BirA^HA^ were tested for statistically significant differences. To this end, ordinary one‐way or two‐way ANOVAs with correction for multiple comparisons were conducted. A corrected mixed‐effects model was used if data were not independently obtained. The used tests are provided in the respective figure captions. Prior to variance analysis, all data sets were tested for normal distribution with the Shapiro–Wilk test (passed if *p* ≥ 0.05) and for equal variances with the Brown‐Forsythe test (passed if *p* ≥ 0.05). For correlation analysis of construct expression levels or microtubule binding affinity versus sorting efficiency, simple linear regression analysis was performed.

### 2.6 Interactome Analysis

#### 2.6.1 Sample Preparation

The sample preparation was adapted from previous studies (Cho et al. 2020) with slight modifications. 200 μg protein lysate was incubated with streptavidin‐coated beads (Thermo Fisher) overnight under rotation at 4°C, washed, and on‐bead digested with 2 M urea in 50 mM Tris–HCl (pH 7.5) with 1 mM dithiothreitol (DTT, Thermo Fisher) and 5 μg/mL trypsin (Thermo Fisher) for 1 h. After reduction with 4 mM DTT for 40 min and alkylation with 10 mM chloroacetamide (CAA) for 50 min in the dark, 0.5 μg trypsin was added for overnight digestion. Digestion was stopped by adding formic acid (final: 1%). The eluate was centrifuged for 5 min at 13,000 g and loaded onto equilibrated SDB‐RP C18 StageTips (Thermo Fisher), which were then washed, centrifuged at full speed to completely dry, and stored at 4°C for subsequent LC–MS analysis.

#### 2.6.2 LC–MS/MS Analysis

Samples were analyzed by the CECAD proteomics facility (CECAD institute, Cologne) on a Q Exactive Plus Orbitrap mass spectrometer that was coupled to an EASY nLC (both Thermo Scientific). Peptides were loaded with solvent A (0.1% formic acid in water) onto an in‐house packed analytical column (50 cm length, 75 μm inner diameter, filled with 2.7 μm Poroshell EC120 C18, Agilent). Peptides were chromatographically separated at a constant flow rate of 250 nL/min using the following gradient: initial 3% solvent B (0.1% formic acid in 80% acetonitrile), from 3% to 5% B within 1.0 min, from 5% to 30% solvent B within 65.0 min, from 30% to 50% solvent B within 13.0 min, from 50% to 95% solvent B within 1.0 min, followed by washing and column equilibration. The mass spectrometer was operated in data‐dependent acquisition mode. The MS1 survey scan was acquired from 300 to 1750 m/z at a resolution of 70,000 and 20 ms maximum injection time. The top 10 most abundant peptides were isolated within a 1.8 Th window and subjected to HCD fragmentation at a normalized collision energy of 27%. The AGC target was set to 5e5 charges, allowing a maximum injection time of 110 ms. Product ions were detected in the Orbitrap at a resolution of 35,000. Precursors were dynamically excluded for 10.0 s.

All mass spectrometric raw data were processed with MaxQuant (version 2.2.0.0, (Tyanova, Temu, and Cox 2016)) using default parameters against the Uniprot canonical human database (UP5640, downloaded 05.08.2020) with the match‐between‐runs option enabled between replicates. Sample normalization was performed as previously described (Cox et al. 2014). Follow‐up analysis was done in Perseus 1.6.15 (Tyanova, Temu, Sinitcyn, et al. 2016). Protein groups were filtered for potential contaminants. Remaining IDs were filtered for data completeness (100% valid values in at least one group) and missing values imputed by sigma downshift (0.3 *σ* width, 1.8 *σ* downshift). Next, all sample groups were filtered for IDs that were enriched (log_10_ (*p*‐value) > 1.3, log_2_ (fold change) > 0.58) compared to both control groups, onlyBirA^HA^ and only 0N3R^HA^. The unfiltered list of all detected proteins and the protein lists after background filtering are available (Table S1a,b). For the remaining IDs, FDR‐controlled two‐sided *t*‐tests were performed between all sample groups.

Gene ontology (GO) term analyses were performed for BirA‐0N3R^HA^ versus BirA‐Nterm^HA^, BirA‐0N4R^HA^ versus BirA‐Nterm^HA^, and BirA‐0N3R^HA^ and BirA‐0N4R^HA^ using gProfiler (http://biit.cs.ut.ee/gprofiler/gost). Detailed outcomes of all gProfiler queries are available (Tables S4–S8). Graphical illustration of interactome and GO results was done with the ggplot2 package (Version: 3.5.1) in RStudio (Version: 2024.04.1) (see Data S1) (Team 2023; Wickham et al. 2019).

## 3 Results

### 3.1 Endogenous‐Like Axonal Sorting of Exogenous 0N3R‐Tau^HA^ in Human *MAPT* ‐KO iPSC‐Neurons

Primary rodent cultures often show poor axonal sorting of exogenous Tau (Iwata et al. 2019; Xia et al. 2016), and human neuron models for axodendritic Tau sorting are scarce. We differentiated human Ngn2‐transgenic WTC11 iPSCs (Miyaoka et al. 2014; Zhang et al. 2013) with and without biallelic *MAPT* knockout (Buchholz et al. 2025) (Figure S1a) into cortical glutamatergic neurons (Buchholz, Bell‐Simons, Cakmak, et al. 2024; Tracy et al. 2022; Wang et al. 2017) to characterize axonal Tau sorting.

**FIGURE 1:**
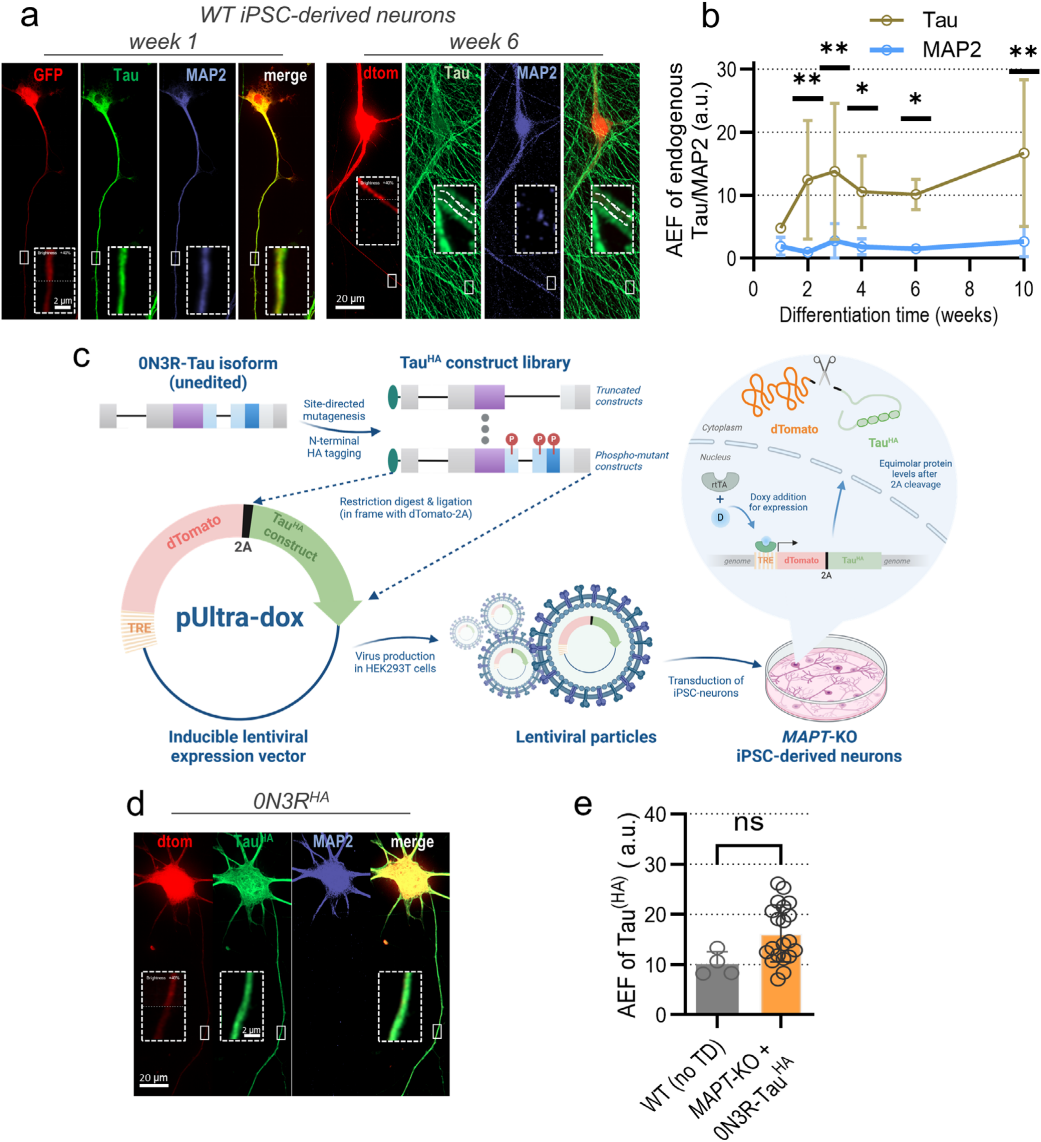
Differentiation of human iPSC‐derived neurons and lentivirus production. (a) Human Ngn2‐transgenic WTC11 WT iPSCs were differentiated into iPSC‐neurons and transduced with lentiviruses containing pUltra/pUltra‐chili vectors for constitutive GFP/dTomato expression at different time‐points post‐differentiation. Cultures were fixed at week 1, 2, 3, 4, 6 and 10 post‐differentiation and immunostained. Signals for Tau (green), MAP2 (blue) and dTomato/GFP (red) are shown in single channels and merged (green + red). Axonal sections (dashed boxes) were magnified. Dashed lines inside the magnifications 6‐week‐old neurons indicate the axon. Scale‐bar: 20 μm, scale‐bar within dashed box: 2 μm. (b) Axonal enrichment factor (AEF) of endogenous Tau and MAP2 during differentiation normalized to dTomato. Quantification from three to five independent experiments (colored dots) included 8–21 cells per experiment. The colored dots indicate the arithmetic mean, the error bars show SD. An ordinary two‐way ANOVA with Tukey's correction determined significance levels between different weeks and between two AEF values of the same week. Significance levels: *p < 0.05, **p < 0.01, ns: *p* ≥ 0.05. (c) Workflow of Tau^HA^ library sorting analysis. The recombinant Tau^HA^ library was generated with site‐directed mutagenesis, with Tau^HA^ constructs cloned into the lentiviral transfer vector pUltra‐dox in frame with the 2A‐coupled reporter dTomato under regulation of a tetracycline response element (TRE). Virus particles were produced in HEK293T cells and transduced to differentiated iPSC‐neurons carrying a biallelic *MAPT*‐KO, resulting in undirected genomic integration. Addition of doxycycline (D) 24 h after transduction leads to binding of the reverse tetracycline‐activated transactivator (rtTA) to the TRE and equimolar expression of Tau^HA^ constructs and dTomato. Figure was created with Biorender. (d) *MAPT*‐KO iPSC‐neurons after expression of 0N3R‐Tau^HA^ for 12 days and subsequent immunostaining. Signals for Tau^HA^ (green), MAP2 (blue), and dTomato (red) are shown in single channels and merged (green + red). Axonal sections are magnified (dashed boxes). Scale bar: 20 μm, scale bar within dashed box: 2 μm. (e) Axonal enrichment factor (AEF) of 0N3R‐Tau^HA^ in *MAPT*‐KO iPSC‐neurons compared to AEF of endogenous Tau in 6‐weeks‐old WT iPSC‐neurons. Quantification from four (WT) or 20 (*MAPT*‐KO) independent experiments (black dots) including 9–21 cells per experiment. The colored bars indicate arithmetic means, the error bars show SD. An unpaired *t*‐test was performed to determine significance levels between both groups. Significance levels: Ns: *p* ≥ 0.05.

First, we quantified Tau sorting in WT iPSC‐neurons at different stages of differentiation using constitutive lentiviral dTomato expression (Figure 1a) as a cellular volume marker due to its unbiased distribution pattern (Figure S1d,e). The axonal enrichment of endogenous Tau in the axon compared to the soma increases strongly after week 1, stabilizes by week 6, and elevates towards week 10 with high replicate variability, while MAP2 shows no axonal enrichment at any age (Figure 1b). Axonal Tau sorting coincides with the detectable formation of the axon initial segment (AIS), indicated by the accumulation of ANKG and TRIM46 (Figure S2), in line with previous studies (Lindhout et al. 2020). Both Tau and MAP2 showed modest dendritic enrichment, with MAP2 being more enriched at week 3 (Figure S1b). Some dendritic Tau presence is expected, as this has been documented previously, and there is evidence for dendrite‐specific functions of Tau under healthy conditions, such as negative regulation of NMDAR‐induced synaptic activity and modulation of spine turnover (Ittner and Ittner 2018; Ittner et al. 2010; Zempel et al. 2013). Both Tau and MAP2 showed little nuclear localization throughout differentiation (Figure S1c).

Next, we checked the axonal sorting efficiency of HA‐tagged 0N3R in *MAPT*‐KO iPSC‐neurons using doxycycline‐inducible lentiviral expression of 0N3R^HA^‐Tau and P2A peptide‐coupled dTomato (Buchholz, Bell‐Simons, Cakmak, et al. 2024) (Figure 1c). Lentiviral particles were transduced into 6‐weeks‐old *MAPT*‐KO iPSC‐neurons at low titers, and doxycycline was added for equimolar expression of 0N3R^HA^ and dTomato (Figure 1c,d). We quantified axonal sorting of 0N3R^HA^ after 12–13 days (Figure 1d) and compared it to endogenous Tau (Figure 1e). Strikingly, 0N3R^HA^ sorted with high efficiency, similar to or even more efficient than endogenous Tau, despite an increase in overall abundance due to robust overexpression (Figure S3h). The dendritic and nuclear enrichment of 0N3R^HA^ was similar to or modestly increased, respectively (Figure S3b,c). Axonal, dendritic, and nuclear MAP2 was not enriched after 0N3R^HA^ overexpression compared to *MAPT*‐KO iPSC‐neurons (Figure S3d–f). The correlation analysis between sorting and protein levels revealed a modest positive correlation (Figure S3g).

**FIGURE 2:**
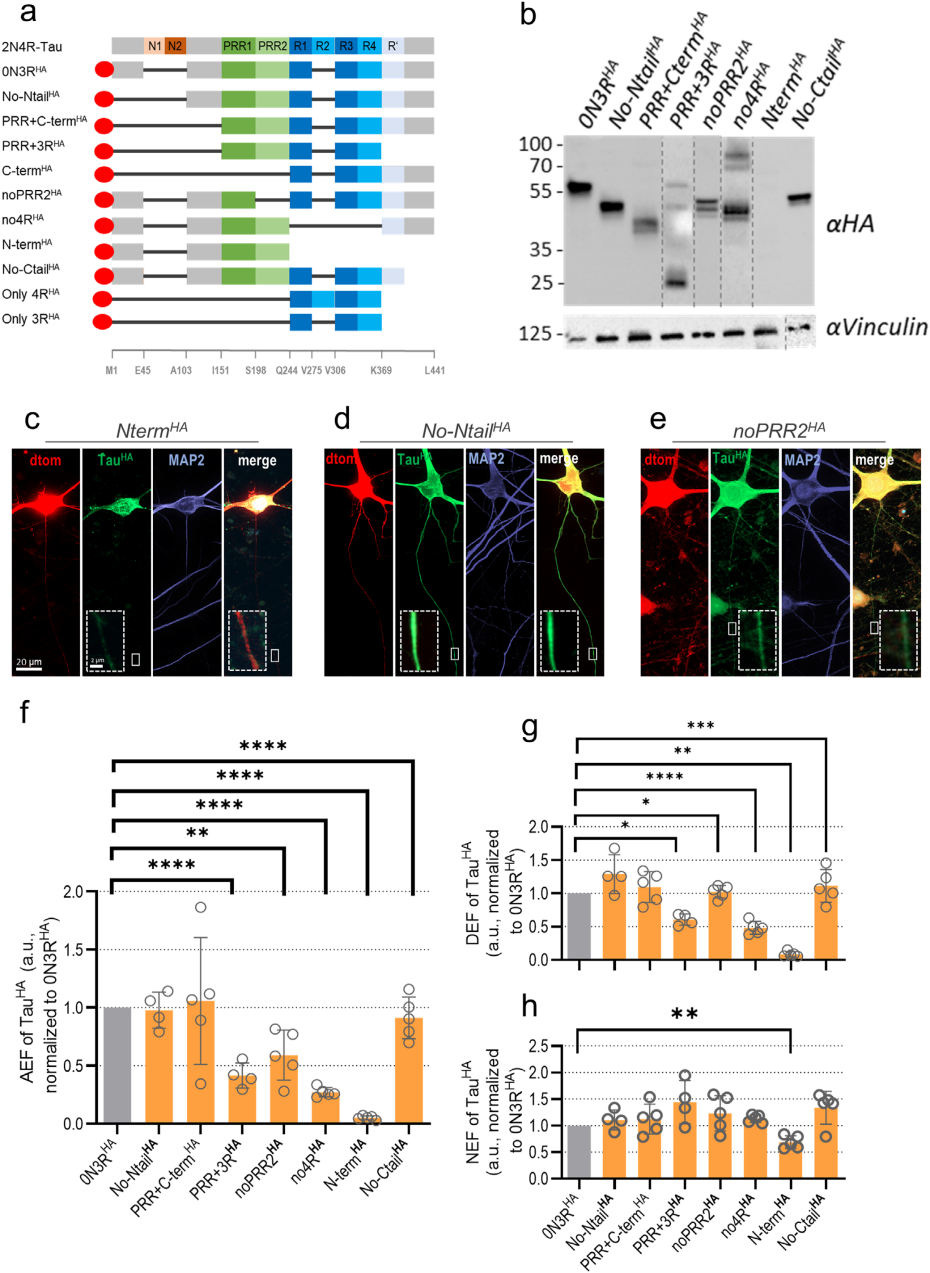
Sorting analysis of transduced 0N3R‐Tau^HA^ and truncated Tau constructs. *MAPT*‐KO iPSC‐neurons were transduced with differently truncated Tau^HA^ constructs at week 6 and harvested after 12–13 days of expression. (a) All Tau^HA^ constructs with domain truncation. The 2N4R‐Tau isoform and selected amino acid positions are shown for orientation. Black bars indicate missing domains, and the red oval indicates the HA tag. Brief construct names are on the left. The constructs Cterm^HA^, only3R^HA^, and only4R^HA^ were excluded from further analysis due to low expression/fast degradation (see Results section). Ntail/Ctail, *N*/C‐terminal tail; Nterm/Cterm, *N*/C‐terminal half; PRR, proline‐rich region; 3R/4R, C‐terminal repeat domains. (b) Western blot analysis for differently transduced *MAPT*‐KO iPSC‐neurons. Vinculin was used as loading control. Protein expression was confirmed for all constructs except for N‐term^HA^, only3R^HA^ and only4R^HA^. Notably, noPRR2^HA^ exhibits two close but distinct bands. The relative abundance of uncleaved dTomato fusion products compared to cleaved constructs is minimal. Lane contrast was adjusted for better visualization (dotted lines indicate crop sites). (c–e) *MAPT*‐KO iPSC‐neurons after expression of Nterm^HA^ (c), No‐Ntail^HA^ (d), or noPRR2^HA^ (e) and dTomato and subsequent immunostaining. Signals for Tau^HA^ (green), MAP2 (blue) and dTomato (red) are shown in single channels and merged (green + red). Axonal sections are magnified (dashed boxes) in merged images. Scale bar: 20 μm, scale bar within dashed box: 2 μm. (f) Axonal enrichment factor (AEF) of truncated Tau^HA^ constructs in KO iPSC‐neurons, normalized to AEF of 0N3R‐Tau^HA^. (g) Dendritic enrichment factor (DEF) of truncated Tau^HA^ constructs in KO iPSC‐neurons after 12–13 days expression, normalized to DEF of 0N3R‐Tau^HA^. (h) Nuclear enrichment factor (NEF) of truncated Tau^HA^ constructs in KO iPSC‐neurons after 12–13 days expression, normalized to NEF of 0N3R‐Tau^HA^. Quantification for (f–h) was done for four to five independent experiments (black dots) with 8–19 cells per experiment. The bars indicate the arithmetic mean, error bars show SD. For all data sets, a mixed‐effects model with Dunnett's correction for multiple comparisons was performed to determine significance levels between 0N3R‐Tau^HA^ and all Tau^HA^ constructs. Non‐normalized ratios of 0N3R‐Tau^HA^ and truncated Tau^HA^ constructs were used for the statistical analysis. Significance levels: *p < 0.05, **p < 0.01, ***p < 0.001, ****p < 0.0001, ns: *p* ≥ 0.05.

Taken together, exogenous 0N3R^HA^ showed endogenous‐like axonal sorting in *MAPT*‐KO iPSC‐neurons without disturbing MAP2 polarization, and we employed this model to study the sorting behavior of truncated and phosphorylation‐mutant Tau^HA^ (Figure 1c; Figure 2a; Table 1).

**TABLE 1:** List of all truncated mutant and phospho‐mutant Tau^HA^ constructs generated with site‐directed mutagenesis.

Category	Constructs
Truncated constructs	0N3R‐Tau^HA^, No‐Ntail‐Tau^HA^, PRR + C‐term‐Tau^HA^, PRR + 3R‐Tau^HA^, C‐term‐Tau^HA^, noPRR2‐Tau^HA^, no4R‐Tau^HA^, No‐Ctail‐Tau^HA^
Phospho‐mutant constructs	S199E‐Tau^HA^, S202E‐Tau^HA^, T205E‐Tau^HA^, AT8 all E‐Tau^HA^, S199A‐Tau^HA^, S202A‐Tau^HA^, T205A‐Tau^HA^, AT8 all A‐Tau^HA^, 1xKXGA‐Tau^HA^, 3xKXGA‐Tau^HA^, 1xKXGE‐Tau^HA^, 2xKXGE‐Tau^HA^, 3xKXGE‐Tau^HA^, AT8‐KXGE‐Tau^HA^, AT8‐KXGA‐Tau^HA^, P301L‐2N4R‐Tau^HA^

### 3.2 Mutant Tau^HA^ Constructs Show Robust Expression Levels and Highly Efficient 2A Cleavage

First, we validated protein expression and 2A cleavage of all mutant Tau^HA^ constructs in *MAPT*‐KO iPSC‐neurons. Unmutated 0N3R^HA^ appeared at the expected size of ~55 kDa; a larger band of uncleaved dTomato‐2PA‐0N3R‐Tau^HA^ was barely visible at ~95 kDa, suggesting highly efficient cleavage of the P2A (Figure 2b; Figure S4a–c; Figure S7f). For most of the truncated Tau^HA^ constructs (Figure 2a), robust expression of correctly sized products was detected, supported by the immunofluorescent data (Figure S4d). For Cterm^HA^, PRR‐3R^HA^, only3R^HA^, and only4R^HA^, very weak or no distinct bands were detectable (Figure S4a–c), in line with decreased protein levels compared to 0N3R‐Tau^HA^ (Figure S4d), indicating posttranslational degradation. All three constructs were thus excluded from further analysis. We observed three distinct bands for noPRR2‐Tau^HA^ that might hint towards variations in phosphorylation or other posttranslational modifications (PTM). Cterm^HA^, PRR + Cterm^HA^ and only4R^HA^ showed slightly increased sorting with higher protein amounts; all other fragments had no detectable correlation (Figure S4e,f).

**FIGURE 3:**
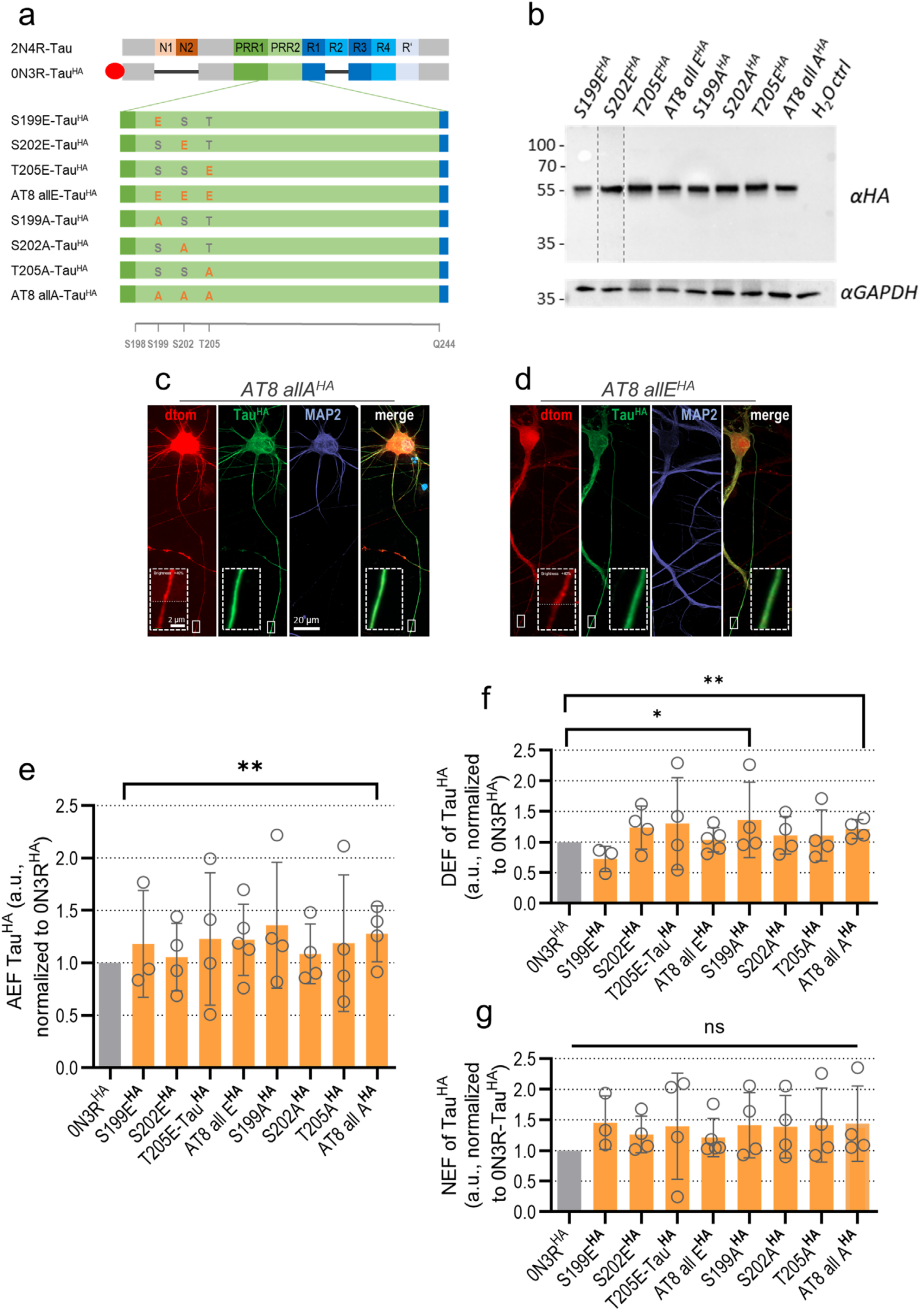
Sorting analysis of transduced 0N3R‐Tau^HA^ and AT8‐mutant Tau^HA^ constructs. *MAPT*‐KO iPSC‐neurons were transduced with different AT8‐mutant Tau^HA^ constructs at week 6 and harvested after 12–13 days of expression. (a) All Tau^HA^ constructs contained pseudo‐phosphorylated (Ser/Thr to Glu) or phosphorylation‐deficient (Ser/Thr to Ala) residues within the AT8 motif. The 2N4R‐Tau isoform and selected amino acid positions are given for orientation (see also Figure S3a). Black bars symbolize the lack of the respective domain, the red oval indicates the HA tag. Brief construct names are indicated on the left. S, Serine; T, Threonine; A, Alanine; E, Glutamic acid. (b) Western blot analysis for differently transduced *MAPT*‐KO iPSC‐neurons, with GAPDH as loading control. All bands of AT8‐mutant Tau^HA^ constructs appear at the expected 55 kDa size. Lane contrast was adjusted for better visualization (dotted lines indicate crop sites, see Figure S7c for raw blots). Protein levels were not quantified due to different transduction efficiencies. (c, d) *MAPT*‐KO iPSC‐neurons co‐expressing either AT8 allA^HA^ (c) or AT8 allE^HA^ (d) with dTomato were immunostained. Tau^HA^ (green), MAP2 (blue) and dTomato (red) are shown in single channels and merged. Axonal sections are magnified (dashed boxes) in merged images. Scale bar: 20 μm, scale bar in magnification: 2 μm. (e) Axonal enrichment factor (AEF), (f) dendritic enrichment factor (DEF), (g) and nuclear enrichment factor (NEF) of AT8‐mutant Tau^HA^ constructs in KO iPSC‐neurons, normalized to 0N3R‐Tau^HA^. Quantification was done for four to five independent experiments (8–19 cells per experiment). Bars indicate the arithmetic mean, error bars show SD. For all data sets, a mixed‐effects model with Dunnett's correction for multiple comparisons was performed to determine significance levels between non‐normalized ratios of 0N3R‐Tau^HA^ and all Tau^HA^ constructs. Non‐normalized ratios of 0N3R‐Tau^HA^ and AT8‐mutant Tau^HA^ constructs were used for the statistical analysis. Significance levels: *p < 0.05, **p < 0.01, ns: *p* ≥ 0.05.

AT8‐mutant Tau^HA^ constructs were all expressed robustly with expected sizes (Figure 3a and Figure S5c). Higher molecular bands of uncleaved fusion protein were negligibly faint suggesting almost complete P2A cleavage (Figure 3b; Figures S5a,b and S7f). Correlation analysis showed a positive correlation of protein levels and axonal sorting for S199E^HA^, T205E^HA^, AT8‐allE^HA^, and AT8‐allA^HA^ (Figure S5d,e).

**FIGURE 4:**
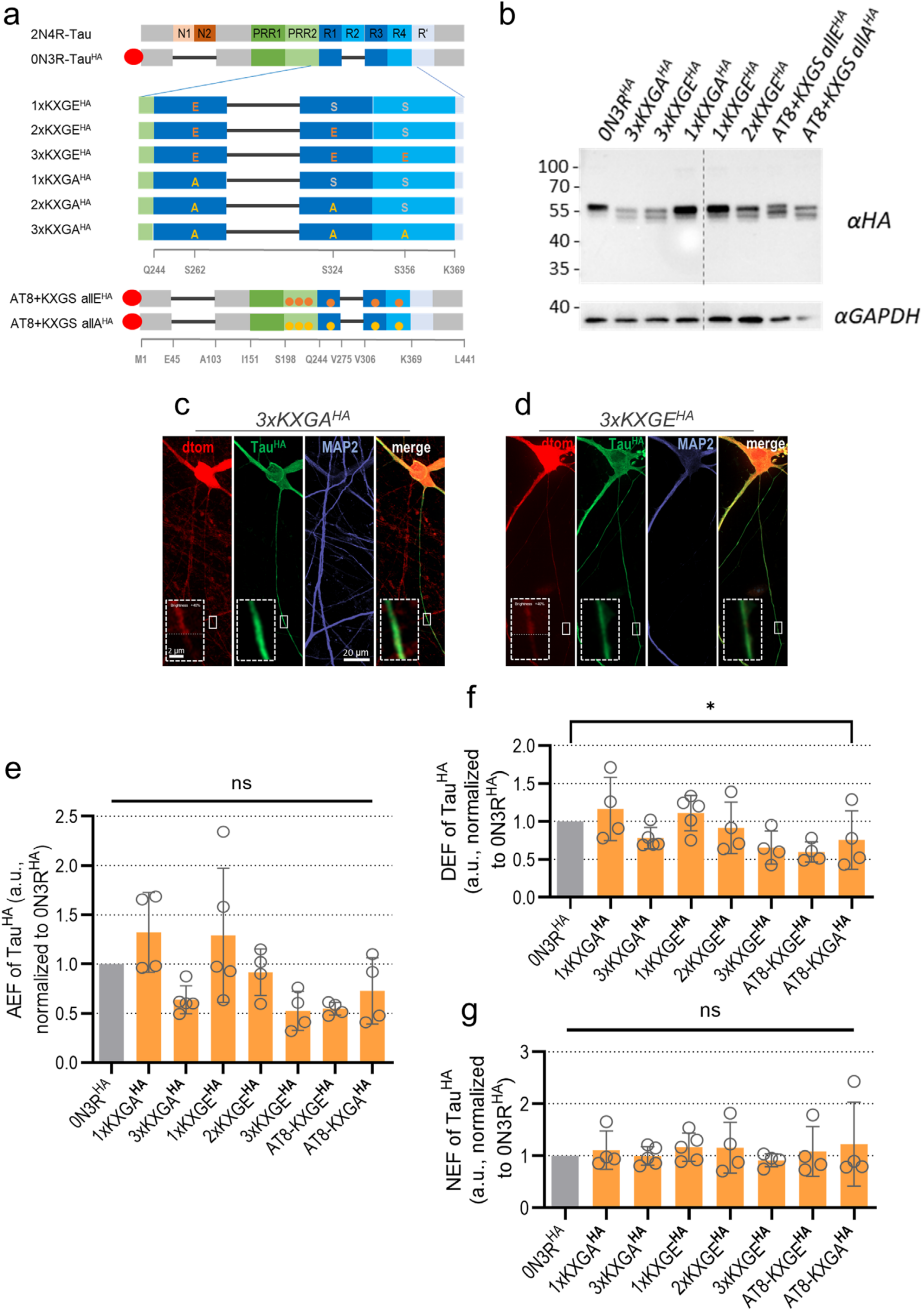
Sorting analysis of transduced 0N3R‐Tau^HA^ and KXGS‐ and double‐mutant Tau^HA^ constructs. *MAPT*‐KO iPSC‐neurons were transduced with different KXGS‐ and double‐mutant Tau^HA^ constructs at week 6 and harvested after 12–13 days of expression. (a) Tau^HA^ constructs included pseudo‐phosphorylated (Ser/Thr to Glu) or phosphorylation‐deficient (Ser/Thr to Ala) residues within the KXGS motif, and double KXGS‐AT8 mutants. The 2N4R‐Tau isoform and selected amino acid positions are displayed for orientation (see also Figure S3a). Black bars symbolize the lack of the respective domain, the red oval indicates the HA tag. Construct names are indicated at the left. S, Serine; T, Threonine; A, Alanine; E, Glutamic acid. (b) Western blot analysis for differently transduced *MAPT*‐KO iPSC‐neurons, using GAPDH as loading control. All bands appeared at the expected 55 kDa size, with distinct bands of varying intensity ratios, possibly due to differential posttranslational modification. Lane contrast was adjusted for better visualization (dotted lines indicate crop sites, see Figure S7c for raw blots). Protein levels were not quantified due to different transduction efficiencies. (c, d) *MAPT*‐KO iPSC‐neurons co‐expressing 3xKXGA^HA^ (c) or 3xKXGE^HA^ (d) with dTomato were immunostained. Tau^HA^ (green), MAP2 (blue) and dTomato (red) signals are shown in single channels and merged. Axonal sections are magnified (dashed boxes). Scale bar: 20 μm, scale bar within dashed box: 2 μm. (e–g) Axonal enrichment factor (AEF), (f) dendritic enrichment factor (DEF), and (g) nuclear enrichment factor (NEF) of KXGS‐ and double‐mutant Tau^HA^ constructs in KO iPSC‐neurons, normalized to 0N3R‐Tau^HA^. Quantification for (f, h) was done for four to five independent experiments (black dots) with 8–19 cells per experiment. The bars indicate the arithmetic mean, error bars show SD. For all data sets, a mixed‐effects model with Dunnett's correction for multiple comparisons was performed to determine significance levels between 0N3R‐Tau^HA^ and all Tau^HA^ constructs. Non‐normalized ratios of 0N3R‐Tau^HA^ and KXGS‐ and double‐mutant Tau^HA^ constructs were used for the statistical analysis. Significance levels: *p < 0.05, ns: *p* ≥ 0.05.

KXGS‐ and double‐mutant Tau^HA^ constructs were expressed robustly (Figure S6d) and detected with distinct double bands around 50 kDa with either roughly equal proportions (3xKXGA^HA^, 3xKXGE^HA^, AT8 + KXGS all E^HA^, AT8 + KXGS all A^HA^) or a dominant larger band (1xKXGA^HA^, 1xKXGE^HA^) (Figure 4b and Figure S6a–c). Higher molecular bands of uncleaved fusion proteins were negligibly faint for all constructs (Figure 4b; Figures S6a–c and S7f).

In brief, the investigated Tau^HA^ constructs were mainly expressed in our *MAPT*‐KO iPSC‐neurons without signs of protein degradation, except for Cterm^HA^, Nterm^HA^, and PRR + 3R^HA^, only3R^HA^, and only4R^HA^, and with highly efficient cleavage of 2A‐coupled dTomato reporter proteins.

### 3.3 Axonal Tau Sorting Requires the PRR2 Domain but Is Independent of the N‐Terminal Tail and Tau Microtubule Affinity

Next, we quantified the sorting efficiency of all truncated Tau^HA^ constructs in relation to 0N3R^HA^‐Tau in *MAPT*‐KO iPSC‐neurons. When the N‐ or C‐terminal tails were missing (No‐Ntail^HA^, No‐Ctail^HA^), we saw efficient axonal sorting while lacking larger parts of the C‐terminus (Nterm^HA^) led to failed axonal enrichment (~5%–20% efficiency of 0N3R^HA^) (Figure 2f). Addition of the PRR domain (PRR + Cterm^HA^) rescued axonal targeting (Figure 2d) but the microtubule‐binding domain alone (PRR + 4R^HA^) was in turn much less abundant in the axon. Significantly decreased axonal sorting was observed when Tau was lacking the repeat domains completely (no4R^HA^) (Figure 2f). Remarkably, the changes in dendritic enrichment of truncated Tau^HA^ constructs followed a similar pattern (Figure 2g). Direct correlation of the axonal and dendritic enrichment (axon/dendrite ratio, Figure S7b) revealed that most constructs had no changes in axon to dendrite enrichment, compared to 0N3R^HA^. Only for noPRR2^HA^ and Cterm^HA^, which showed decreased protein levels (Figure S4d), the axon/dendrite ratio was significantly decreased, indicating axon‐specific impairments of intracellular sorting for those constructs (Figure S7b). The nuclear levels were not changed for any truncated Tau^HA^ construct except for Nterm^HA^, likely due to the somatic inclusion formation (Figure 2h).

We tested the correlation between sorting efficiency and microtubule affinity, which was determined by Tau‐tubulin binding assays (Gustke et al. 1992; Gustke et al. 1994) (Figure S7e), and found that the axonal enrichment increased with higher microtubule affinity (Figure S7c), in contrast to earlier findings (Iwata et al. 2019). We also observed a positive correlation for the axon/dendrite ratio (Figure S7d). This indicates that microtubule affinity is essential for axonal, but also for dendritic enrichment.

For the AT8‐mutant Tau^HA^ constructs, axonal sorting was equally efficient for all constructs with pseudophosphorylation or dephosphorylation. Only the phospho‐dead AT8‐allA^HA^ construct was slightly higher axonally enriched than 0N3R^HA^ (Figure 3e). The AT8‐allA^HA^ construct and S199A^HA^ were also modestly increased in dendrites (Figure 3f). The nuclear enrichment was not changed for any of the AT8‐mutant Tau^HA^ constructs (Figure 3g).

For our KXGS‐ and double‐mutant Tau^HA^ constructs, axonal sorting efficiency gradually declined from 1xKXGA^HA^ to 3xKXGA^HA^ and from 1XKXGE^HA^ to 2xKXGE^HA^ to 3XKXGE^HA^ (Figure 4e), but without reaching statistical significance. The axon/dendrite ratio was unchanged for all constructs (Figure S7b) as dendritic enrichment was similar to the axonal pattern (Figure 4f). Axonal targeting of double mutants was similar to KXGS^HA^ mutants, in line with the missing effect of AT8 single mutants (Figure 3e). The nuclear enrichment of all KXGS‐ and double‐mutant Tau^HA^ constructs was not different from 0N3R^HA^ (Figure 4g).

**FIGURE 5:**
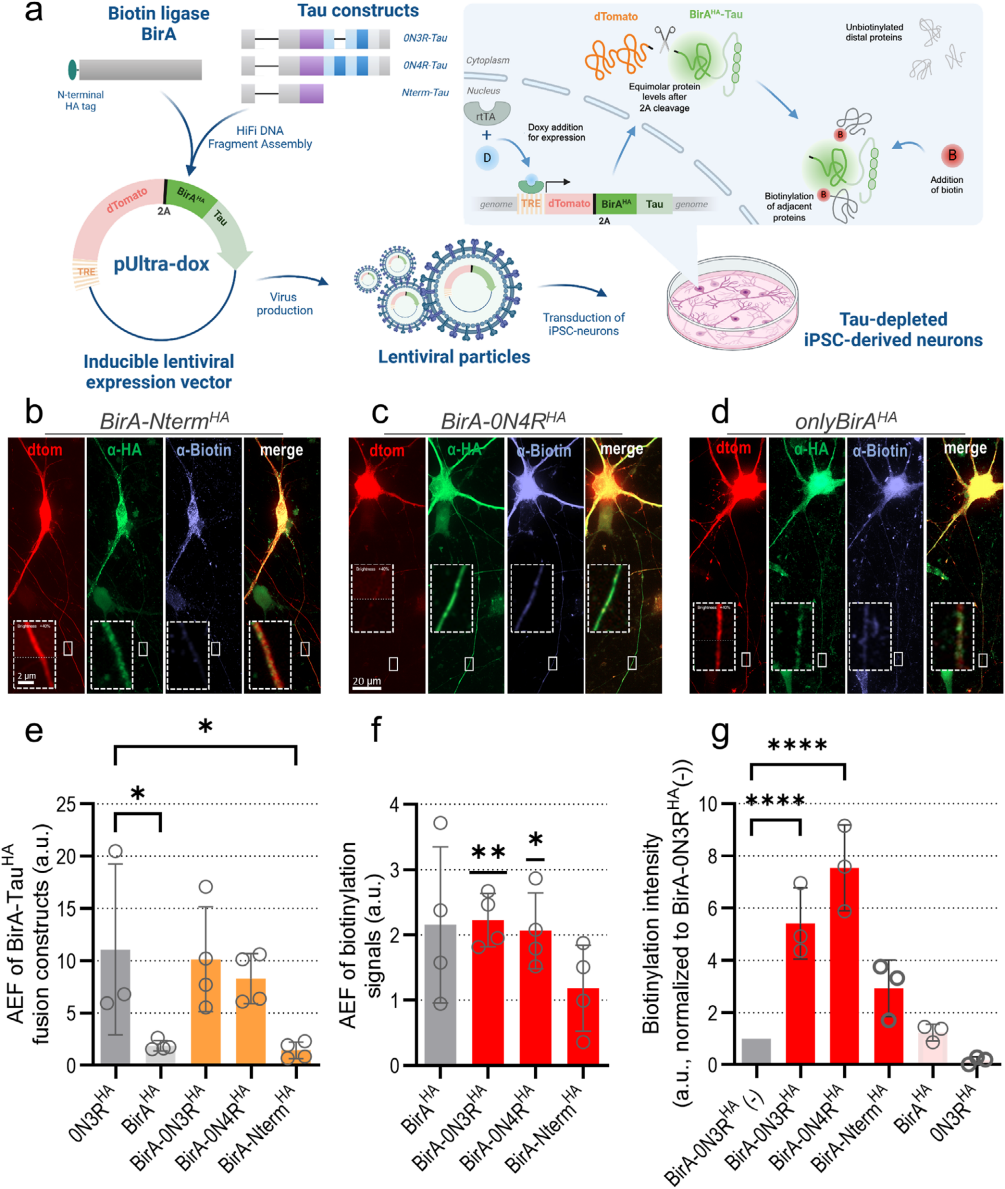
Generation and characterization of BirA‐Tau fusion constructs. *MAPT*‐KO iPSC‐neurons were transduced with BirA‐Tau^HA^ constructs at week 6 and biotinylated followed by harvesting after 12–13 days of expression. (a) Workflow of BirA‐Tau^HA^ fusion construct generation. Coding sequences of BirA^HA^ and the Tau isoforms (0N3R, 0N4R or Nterm‐Tau) were cloned into the lentiviral transfer vector pUltra‐dox with a 2A‐coupled dTomato reporter, regulated by a tetracycline response element (TRE). Virus particles were harvested from HEK293T cells, and transduced to differentiated *MAPT‐KO* iPSC‐neurons (see Figure S1a). Doxycycline addition 24 h after transduction leads to binding of the reverse tetracycline‐activated transactivator (rtTA) to the TRE and equimolar expression of BirA‐Tau^HA^ fusion constructs and the reporter protein dTomato. 20 min prior to fixation, biotin was added to induce biotinylation of adjacent proteins by BirA. Figures was created with Biorender. (b–d) *MAPT*‐KO iPSC‐neurons expressing BirA‐Nterm^HA^ (c), BirA‐0N4R^HA^ (d), and onlyBirA^HA^ (e) and dTomato were immunostained. BirA‐Tau^HA^ or BirA^HA^ (green), biotin (blue) and dTomato (red) signals are shown in single channels and merged. Axonal sections are magnified (dashed boxes). Scale bar: 20 μm, scale bar within dashed box: 2 μm. (e) Axonal enrichment factor (AEF) of BirA‐Tau^HA^ fusion proteins, compared to the AEF of 0N3R‐Tau^HA^ and BirA^HA^. Quantification from three to four experiments (black dots) included 8–18 cells per experiment. Bars indicate the arithmetic mean, error bars show SD. A mixed‐effects model with Dunnett's correction for multiple comparisons was performed to determine significance levels between the groups. Significance levels: *p < 0.05, ns: *p* ≥ 0.05. (f) Axonal enrichment factor (AEF) of biotin after expression of different BirA‐Tau^HA^ fusion proteins i, compared to AEF of biotin in neurons expressing only BirA^HA^. Quantified from 3 to 4 experiments (black dots) (8–18 cells per experiment). Bars indicate the arithmetic mean, error bars show SD. Groupwise one‐sample t‐tests were performed to determine the difference from undirected distribution (AEF = 1). Significance levels: *p < 0.05, **p < 0.01, ns: *p* ≥ 0.05. (g) Biotinylation intensity after expression of BirA‐Tau^HA^ fusion proteins, normalized to the signals in neurons expressing BirA‐0N3R‐Tau^HA^ without biotin treatment. Quantified from 3 to 5 experiments (black dots) (8–18 cells per experiment). Bars indicate the arithmetic means, error bars show SD. A mixed‐effects model with Dunnett's correction for multiple comparisons was performed to determine significance levels between the groups. Non‐normalized values were used to perform statistical analysis. Significance levels: *p < 0.05, **p < 0.01, ****p < 0.0001, ns: *p* ≥ 0.05.

Of note, the intracellular sorting behavior of endogenous MAP2 was not changed upon expression of any truncated or AT8/KXGS‐modified Tau^HA^ construct (Figure S3g–i; Figures 4f–h and 5g–i) indicating that expression of Tau, even though also markedly present in the dendrites, did not displace MAP2.

Taken together, we found that the absence of the PRR2 domain leads to axon‐specific Tau sorting deficits, and that microtubule affinity was not linked to impaired axonal sorting in both truncated and phospho‐mutant Tau^HA^ constructs.

### 3.4 TurboID Proximity Labeling Is Suitable for Analyzing Axonal Binding Partners of BirA‐Coupled Tau^HA^

Next, we studied Tau interactions in *MAPT*‐KO iPSC‐neurons by using biotinylation‐based TurboID proximity labeling (Branon et al. 2018; Cho et al. 2020). To unravel isoform‐specific interactors, we coupled the Tau isoforms 0N3R and 0N4R to the biotin ligase BirA. The non‐sorting construct Nterm^HA^ was coupled to detect sorting‐specific Tau binding partners.

First, we successfully confirmed the proper expression of all BirA‐Tau^HA^ constructs (Figure S10a) and efficient 2A‐based cleavage from dTomato (Figure S8a,b). The axonal sorting of BirA‐0N3R^HA^ and BirA‐0N4R^HA^ was similar to that of uncoupled 0N3R^HA^ in immunostained neurons, while there was, as expected, no axonal sorting of the control constructs BirA^HA^ and BirA‐Nterm^HA^ (Figure 5b–e). For BirA‐Nterm^HA^, we observed the puncta‐like distribution typical for Nterm^HA^ (Figure 5b, see also Figure 2c) (Bell et al. 2021). The chosen labeling time ensured sufficient axonal penetration of biotin (Figure 5c,f) without excessive unspecific labeling (Cho et al. 2020). Expression of BirA‐0N3R^HA^ without biotin application revealed only basal biotinylation by BirA (Figure 5g), and correlation analysis of sorting efficiency and protein levels was negative for all BirA‐Tau^HA^ constructs (Figure S8c). Relative protein levels were similar for all BirA‐Tau^HA^ constructs (Figure S7d).

All in all, we could validate the expression, cleavage, and efficient axonal sorting of our BirA‐Tau^HA^ constructs, thus being suitable for Tau interaction analysis.

### 3.5 Key Regulators of Pre‐ and Postsynaptic Plasticity Bind Specifically to BirA‐0N4R^HA^

**FIGURE 6:**
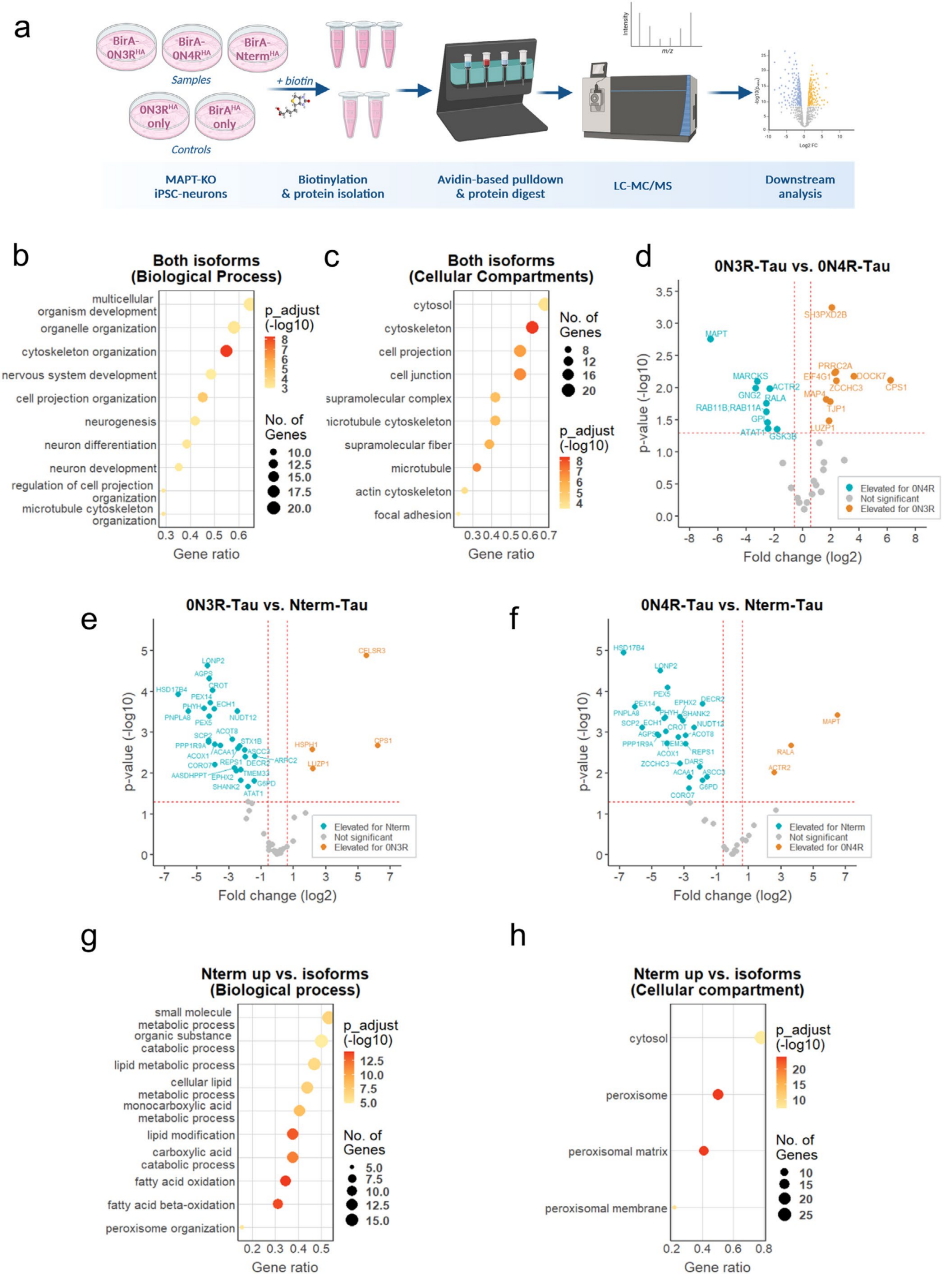
TurboID‐based interactomics study of 0N3R‐ & 0N4R‐Tau and non‐sorting Nterm‐Tau. (a) Workflow for interactomics sample preparation. *MAPT*‐KO iPSC‐derived neurons were differentiated and transduced with BirA‐Tau^HA^ constructs (see Figure 5a and Figure S1a). Neurons were exposed to 500 μM biotin for 20 min, lysed, and biotinylated proteins were enriched with avidin‐coated magnetic beads. Enriched proteins were digested and analyzed by LC–MS/MS. Data were used for downstream interactome analyses. Figure was created with Biorender. (b, c) GO term analysis of 0N3R and 0N4R interaction partners, filtered for background targets, performed with gProfiler. GO terms for biological process (b) and cellular compartment (c) are sorted by the number of matched genes. Dot size represents the number of genes, and color indicates the adjusted *p*‐value. (d) Interactome comparison of BirA‐0N3R^HA^ versus BirA‐0N4R^HA^ based on *p*‐values (−log_10_) and fold‐changes (log_2_) of label‐free quantification (LFQ) after filtering of background targets. Proteins enriched for 0N3R‐Tau (thresholds: *p*‐value > 1.3, fold‐change ≤ 0.58) are marked blue, proteins enriched for 0N4R‐Tau (thresholds: *p*‐value > 1.3, fold‐change > 0.58) are marked orange. Gene IDs of different interactors are given. (e, f) Interactome comparison of BirA‐0N3R^HA^ versus BirA‐Nterm^HA^ (e) and BirA‐0N4R^HA^ versus BirA‐Nterm^HA^ (f) with *p*‐values (−log_10_) and fold‐changes (log_2_) of LFQ, after filtering background targets. Proteins enriched for 0N3R‐Tau or 0N4R‐Tau (thresholds: *p*‐value > 1.3, fold‐change ≤ 0.58) are marked blue, proteins enriched for Nterm‐Tau (thresholds: *p*‐value > 1.3, fold‐change > 0.58) are marked orange. Gene IDs of different interactors are given. (g, h) GO term analysis of genes enriched as BirA‐Nterm^HA^ binding partners, based on (e, f), using gProfiler. GO terms for biological process (g) and cellular compartment (h) are sorted by matched genes, with dot size indicating gene count and color indicating adjusted *p*‐values.

For interactome analysis, biotinylated proteins were enriched with avidin‐coated beads, digested, and analyzed with liquid chromatography–coupled mass spectrometry (LC–MS/MS) (Figure 6a).

We detected 744 proteins in our BirA‐Tau^HA^ constructs (Table S1a), of which 62 proteins were significantly enriched for at least one construct compared to both control constructs, onlyBirA^HA^ and only0N3R^HA^ (Table S1b). We compared the identified interactors of BirA‐0N3R^HA^ and BirA‐0N4R^HA^ (33 out of 62 proteins) with data from a study using Tau‐APEX2 in the same parental iPSC line (Tracy et al. 2022), and with data from a human Tau interactome meta‐analysis (Kavanagh et al. 2022). We found an overlap of 23 proteins (69.7%) and 24 proteins (72.7%), respectively (Table 2). A gene ontology (GO) terms analysis of all 33 isoform‐related hits (Tables S7 and S8) revealed a strong association with biological processes such as microtubule function, cytoskeletal protein binding and organization, or nucleotide and ribonucleotide binding (Figure 6b,c), matching with the known major interaction partners of Tau (Guo et al. 2017; Kavanagh et al. 2022). In sum, we confirmed the successful enrichment of Tau‐interacting proteins in our experimental setup.

**TABLE 2:** List of all genes that were found to interact either with BirA‐0N3R^HA^, for BirA‐0N4R^HA^, or with both isoforms. The Uniprot ID and Protein name are given for all genes. Identical gene and protein names are indicated by ‘*’. 0N3R/0N4R interactor: ‘YES’ means that the gene was found to interact in our study, ‘—’ means that no interaction was found. Found in Kavanagh et al. (2022): ‘YES’ means that the protein was described as a Tau interactor in the meta‐analysis of seven human Tau interactome studies (Kavanagh et al. 2022), ‘—’means that it was not described in Kavanagh et al. (2022). The number of hits refers to how many of the seven human Tau interactome papers reported Tau interaction of the protein. Found in Tracy et al. (2022): ‘YES’ means that Tau interaction was found in Tracy et al. (2022) using biotinylation labeling in human iPSC‐neurons, ‘YES*’ means that Tau interaction was not found in Tracy et al. (2022) using biotinylation labeling but FLAG‐based IP of WT‐Tau. ‘—’ means that the protein was not found in Tracy et al. (2022).

Gene	Uniprot ID	Protein	0N3R interactor	0N4R interactor	Found by Kavanagh et al. (2022)	# of hits (Kavanagh et al. 2022)	Found by Tracy et al. (2022)
*DCX*	*O43602*	*	YES	YES	YES	1/7	YES
*MAP2*	*P11137*	*	YES	YES	YES	3/7	YES
*CAPZB*	*P47756*	*	YES	YES	YES	2/7	YES*
*MACF1*	*Q9UPN3*	*	YES	YES	—	—	—
*CASK*	*O14936*	*	YES	YES	YES	1/7	YES*
*LUZP1*	*Q86V48*	*	YES	—	—	—	—
*MAP4*	*P27816*	*	YES	—	YES	4/7	YES
*CPS1*	*P31327*	CPSM	YES	—	YES	1/7	—
*PRRC2A*	*P48634*	PRC2A	YES	—	—	—	—
*EIF4G1*	*Q04637*	IF4G1	YES	—	YES	3/7	YES*
*TJP1*	*Q07157*	ZO1	YES	—	YES	1/7	YES*
*SH3PXD2B*	*A1X283*	SPD2B	YES	—	—	—	—
*MINK1*	*Q8N4C8*	*	YES	—	—	—	—
*DOCK7*	*Q96N67*	*	YES	—	—	—	—
*HSPH1*	*Q92598*	HSP110	YES	—	YES	3/7	YES*
*FERMT2*	*Q96AC1*	FERM2	YES	—	—	—	—
*HN1*	*Q9UK76*	JUPI1	YES	—	YES	1/7	YES*
*EML4*	*Q9HC35*	EMAL4	YES	—	YES	1/7	YES*
*ZCCHC3*	*Q9NUD5*	ZCHC3	YES	—	—	—	—
*CELSR3*	*Q9NYQ7*	CELR3	YES	—	—	—	—
*GPI*	*P06744*	G6PI	—	YES	YES	3/7	YES
*MAPT*	*P10636*	Tau	—	YES	YES	5/7	YES
*RALA*	*P11233*	*	—	YES	YES	1/7	YES*
*ACTR2*	*P61160*	ARP2	—	YES	YES	1/7	YES*
*GSK3B*	*P49841*	GSK‐3β	—	YES	YES	2/7	YES*
*MARCKS*	*P29966*	*	—	YES	YES	2/7	YES*
*ZC2HC1A*	*Q96GY0*	ZC21A	—	YES	YES	1/7	YES*
*GNG2*	*P59768*	GBG2	—	YES	YES	1/7	YES*
*RAB11A*	*P62491*	*	—	YES	YES	1/7	YES*
*RAB11B*	*Q15907*	*	—	YES	YES	1/7	YES*
*ATAT1*	*Q5SQI0*	*	—	YES	YES	1/7	YES*
*KIF21A*	*Q7Z4S6*	KI21A	—	YES	YES	2/7	YES

Next, we determined isoform‐specific interactors out of the 33 proteins (Figure 6d; Figure S11g; Tables S2 and S9). We found nine 0N3R‐specific binding partners, of which three were either directly, LUZP1 and ZO1, or indirectly, SPD2B, linked to the f‐actin cytoskeleton. The 0N3R interactor MAP4 is a microtubule‐binding protein that is structurally related to Tau as both are members of the same MAP family (Nishida et al. 2023; Tokuraku et al. 2010).

Out of the 10 specific interactors of 0N4R, at least 5 proteins are connected to pre‐ or postsynaptic plasticity, vesicle release, and neurite growth, namely the GTPases RALA, RAB11A, RAB11B, the signaling protein MARCKS, and the synaptic serine/threonine kinase GSK‐3β (Arendt et al. 2016; Brudvig et al. 2018; Lalli 2009; Lalli and Hall 2005; Sultana and Novotny 2022; Xu et al. 2014; Zempel and Mandelkow 2014). MARCKS and GSK‐3β are both linked to CDC42 (Brudvig et al. 2018; Zhu et al. 2023), a key regulator of postsynaptic f‐actin plasticity that appears upregulated in AD patients (Zhu et al. 2023). MARCKS also controls vesicle docking in the active zone (Xu et al. 2014). The 0N4R interactors RALA and RAB11A/B affect presynaptic function as they interact with the exocyst complex, a key complex of polarized exocytosis in eukaryotic cells (Lalli 2009; Lalli and Hall 2005; Sultana and Novotny 2022). The remaining 0N4R‐specific interactors were either linked to cytoskeletal functions, such as the microtubule acetyltransferase ATAT1 and the f‐actin‐organizing protein ARP2, or to metabolic function (G6PI).

### 3.6 The Non‐Sorting BirA‐Nterm^HA^ Shows Peroxisomal Association While CELR3 and HSP110 Are Interactors Specific for Sorting BirA‐Tau^HA^

Next, we compared the isoform interaction partners with those of BirA‐Nterm^HA^, which does not show axonal enrichment (Figure 6e,f; Tables S10 and S11). The analysis revealed several specific interactors of BirA‐Nterm^HA^ and a few proteins specifically binding either Tau isoform (Table S3). In the GO term analysis of the Nterm‐specific proteins (Tables S4–S6) the overwhelming number of terms were linked to the cellular compartments peroxisomal membrane and peroxisomal matrix, and to processes including lipid catabolism, fatty acid oxidation, and related peroxisomal processes (Figure 6g). Two proteins of the PTS1‐associated import complex (PEX5, PEX14) were among the interactors. These findings suggest peroxisomal localization of the puncta‐like inclusions of Nterm^HA^ (Figures 2c and 5b). The mechanism of peroxisomal Nterm^HA^ import remains unclear since *in silico* analysis did not predict PTS1‐ or PTS2‐directed import (Brocard and Hartig 2006; Lametschwandtner et al. 1998; Neuberger et al. 2003a, 2003b; Purdue and Lazarow 2001), (PTS1 predictor: https://mendel.imp.ac.at/pts1/, PSORTII: https://psort.hgc.jp/form2.html).

Out of the proteins enriched for the Tau isoforms (Table S3), the heat‐shock protein HSP110 is known to interact with Tau and the major axonal Tau phosphatase PP2A (Eroglu et al. 2010; Kavanagh et al. 2022). When we inhibited PP2A with okadaic acid, we observed elevated levels of total Tau and AT8‐phosphorylation in the soma (Figure S8e–g), suggesting decreased axonal Tau targeting upon PP2A inhibition. Taken together, the sorting‐specific Tau interaction partners are involved in axon guidance and modulation of the axonal Tau‐targeting phosphatase PP2A, while the data hint at the import and accumulation of BirA‐Nterm^HA^ inside the peroxisomal lumen.

## 4 Discussion

Somatodendritic missorting is an early sign of Tau pathology in AD and related diseases (Braak and del Tredici 2011; Braak et al. 2011) but it remains elusive how axonal Tau sorting is maintained in physiology and how axonal Tau sorting is disrupted in disease conditions. Here, we identify Tau domains and cellular interaction partners that are required for efficient axonal Tau sorting and sorting‐specific Tau interaction partners hinting at isoform‐specific roles in normal neuronal function and in disease development.

### 4.1 Correct Expression of Truncated and Phosphorylation‐Mutant Tau Constructs in Human MAPT‐KO iPSC‐Derived Neurons

In our study, we overcame limitations of previous studies (Janke et al. 1999; Kavanagh et al. 2022; Xia et al. 2016) by using rapidly differentiated human‐derived neurons depleted of endogenous Tau and by achieving efficient sorting of overexpressed Tau upon lentiviral delivery. The randomly distributed dTomato, expressed on the same vector but not fused to the Tau constructs due to a 2A cleavage site between the proteins, was used as a reference marker for cellular sorting efficiency.

We could confirm that our Tau^HA^ constructs were expressed correctly after lentiviral transduction. For all constructs, levels of Tau^HA^‐dTomato fusion proteins were negligible, indicating successful cleavage of the 2A peptide, as reported previously (Szymczak‐Workman et al. 2012). Only the heavily truncated constructs only3R^HA^, only4R^HA^ and Cterm^HA^ were not detected in both assays. Since we could exclude problems with virus production or transduction efficiency due to dTomato co‐expression, the lack of detection results either from rapid degradation, e.g., via proteasomal degradation or recognition by another protein quality control pathway reportedly involved in Tau degradation (Amm et al. 2014; Pohl and Dikic 2019; Ruggiano et al. 2014). Notably, the KXGS‐mutant Tau^HA^ constructs exhibited a slightly smaller but distinct additional protein species that was more abundant for constructs with more mutated residues. Disturbed interaction with KXGS‐targeting enzymes such as MARK or PP2A (Chudobová and Zempel 2023; Gong et al. 2000) could affect the posttranslational state of adjacent residues. The Tau^HA^ mutants could also become targets of proteolytic cleavage, which is known to produce similarly sized fragments under pathological conditions (Corsetti et al. 2008; Corsetti et al. 2015; Park and Ferreira 2005).

### 4.2 Axonal Tau Sorting Is Independent of the N‐Terminal Tail, the C‐Terminal Repeat Domains, and the General Microtubule Affinity

In our sorting analysis, we observed poor axonal sorting of both individual protein halves, suggesting that both N‐terminal and C‐terminal parts of Tau are required for axonal sorting, or the AIS as main axonal traffic checkpoint (Leterrier 2016, 2018; Rasband 2010) prevents undisturbed axonal transit. For the N‐terminal half, we confirmed findings from human SH‐SY5Y‐derived neurons and mouse primary neurons showing somatic puncta‐like inclusions (Bell et al. 2021). Of note, some constructs (e.g., PRR + 3R‐Tau or no4R‐Tau) showed changes in both axonal and dendritic sorting, hinting at confounding processes independent of axon‐specific targeting. The considerable dendritic Tau enrichment could be caused by maturation deficits or by technical difficulties of single‐dendrite measurements in dense neuronal cultures.

Previous studies with GFP‐coupled Tau demonstrated that Tau lacking the N‐terminal tail failed to interact with the axonal annexins A2 and A6 and was abnormally redistributed to the soma (Gauthier‐Kemper et al. 2018). However, we observed efficient axonal sorting in the absence of the N‐terminal tail. As we determined the net axonal enrichment instead of only retrograde protein flux (Gauthier‐Kemper et al. 2018), the results suggest that anterograde transport can compensate for retrograde retention deficits. Data from KXGS‐mutant Tau in primary rodent neurons (Li et al. 2011; Zempel et al. 2017) corroborate our findings.

When Tau gets missorted in early AD stages, the KXGS and AT8 residues appear highly phosphorylated (Braak and del Tredici 2011; Islam et al. 2025; Regalado‐Reyes et al. 2019; Zheng‐Fischhöfer et al. 1998). These motifs are crucial for MT binding, which hints at a potential role of changed MT affinity in the early missorting cascade. A recent study with PRR2‐mutant Tau claimed that tight MT binding affinity of Tau impairs axonal sorting as PRR2 phosphorylation‐dead Tau failed to enrich axonally (Iwata et al. 2019). Our results with highly diffusible phosphorylation‐mimetic AT8‐ and KXGS mutants, and previous studies in rodent neurons seem to support this notion (Iwata et al. 2019; Zempel et al. 2017). However, the phosphorylation‐dead AT8 and KXGS mutants did not show any sorting deficits in our *MAPT*‐KO iPSC‐derived neurons. This contradicts the relevance of MT affinity in general. The marginal decrease in bound MTs for PRR2 phosphorylation‐dead mutant Tau cast further doubts in this respect (Iwata et al. 2019). And while depletion of the entire PRR2 domain, thus decreasing its MT affinity (Gustke et al. 1994; Iwata et al. 2019), resulted in poor axonal sorting, depletion of the C‐terminal repeat domains led to less MT binding but no changes in axonal Tau sorting in our and previous studies (Iwata et al. 2019).

Taken together, our results suggest that loss of MT binding affinity, due to hyperphosphorylation of KXGS, AT8, or other motifs, does not cause somatodendritic missorting, as it is observed in early stages of AD. In contrast, changes in phosphorylation and MT affinity may be evoked by cellular responses downstream of the initial missorting trigger.

### 4.3 Axonal Tau Sorting Depends on the PRR2 Domain Independent of AT8 Phosphorylation

Our findings give, together with former studies, a clear picture: Highly diffusible Tau can be sorted efficiently (KXGE mutants, 4R‐lacking construct) (Iwata et al. 2019; Zempel et al. 2017) or inefficiently (PRR2‐lacking, our study, (Iwata et al. 2019)), and also MT‐affine Tau can be sorted efficiently (KXGA mutants, AT8 Ser‐ > Glu mutants, our study) or inefficiently (PRR2 Ser‐ > Glu construct) (Iwata et al. 2019). Thus, we postulate that both the C‐terminal repeat domains and the MT affinity of Tau play no key role in the axonal Tau sorting process.

In contrast, certain residues or motifs of the PRR2 domain appear critical for the axonal sorting of Tau as underlined by the Tau sorting failure of PRR2 dephosphorylation‐mimetic Tau mutants (Iwata et al. 2019). The unaffected sorting of all AT8 mutants makes this motif unlikely to be critical. The PRR was shown to contribute to the MT‐binding geometry of Tau by binding the outer tubulin surface (Amos 2004; Kar et al. 2003). These results question the idea that hyperphosphorylation of the AT8 and KXGS motifs, hallmarks of pathological Tau missorting (Zempel and Mandelkow 2014), is causative for the abnormal cellular localization. Our results rather hint at hyperphosphorylation of AT8 and KXGS residues as a consequence of somatodendritic missorting.

### 4.4 0N4R‐Tau Interacts With Regulators of Synaptic Plasticity That Are Involved in AD Pathogenesis

The human Tau isoforms differ in their intracellular localization (Bachmann, Bell, et al. 2021; Zempel et al. 2017), and studies in rodent neurons hint at isoform‐specific interactors and functions (Liu et al. 2016). Comparable data are lacking for human Tau, although there is evidence for differential roles of Tau isoforms in health and disease conditions (Buchholz et al. 2025; Buchholz and Zempel 2024).

We aimed to identify specific interactors of the 0N3R‐Tau and 0N4R‐Tau, two isoforms with similar cellular distribution (Bachmann, Bell, et al. 2021; Zempel et al. 2017), in *MAPT*‐KO iPSC‐derived neurons using TurboID proximity labelling (Branon et al. 2018; Cho et al. 2020). BirA coupling did not impair the axonal sorting efficiency, and the interactome uncoupled Tau and APEX2‐bound Tau was shown to be consistent (Kavanagh et al. 2022; Tracy et al. 2022), indicating the suitability of biotin‐based Tau interactomics. In our study, the protein number was notably smaller than reported for Tau‐APEX2 constructs (Tracy et al. 2022), probably due to our exclusion of background hits found in control constructs. Still, our GO term analysis and the strong hit consistency with previous studies (Kavanagh et al. 2022; Tracy et al. 2022) demonstrated the validity of our data sets (see Table 2 for comparison of our data to previous studies).

We found evidence for differential isoform effects on MTs, namely 0N3R‐Tau interacted with MAP4, which controls MT branching in neurons (Nishida et al. 2023; Tokuraku et al. 2010) while 0N4R‐Tau was associated with the MT acetyltransferase ATAT1 (Iuzzolino et al. 2024; Li and Yang 2015). This suggests that 0N4R‐Tau promotes MT stability while 0N3R‐Tau rather enhances plasticity, in line with the previous claim of labile plasticity being the key benefit of Tau‐MT binding (Qiang et al. 2018). However, overexpression of individual isoforms in SH‐SY5Y cells did not result in changed MT stability or growth (Bachmann, Bell, et al. 2021), but evidence from more relevant human neuronal cell models is missing.

The C‐terminal repeat domains mediate f‐actin interactions of Tau (Correas et al. 1990; Elie et al. 2015). Accordingly, we saw different f‐actin‐related proteins for 0N3R‐Tau (LUZP1, ZO1) and 0N4R‐Tau (ARP2, MARCKS). The 0N4R‐specific signaling protein MARCKS regulates, besides other cellular functions like cell motility, adhesion, or phagocytosis (Huber et al. 2022; Rodriguez Pena et al. 2013), dendrite growth and spine formation via activation of the CDC42 pathway (Brudvig et al. 2018). Pathological CDC42 overactivation, as observed in AD patients, can induce f‐actin breakdown, synapse defects, and Tau hyperphosphorylation via recruitment of the Tau kinase GSK‐3β (Ying et al. 2022; Zhu et al. 2023). Strikingly, we also identified GSK‐3β, a crucial downstream effector of CDC42 (Zhu et al. 2023), as a 0N4R‐specific interactor, although it targets the PRR2 and C‐terminal tail domains of Tau (Zempel and Mandelkow 2014) but not its repeat domains. These findings suggest that 0N4R‐Tau acts on both upstream regulation of CDC42‐mediated postsynaptic function and downstream effects of CDC42‐induced AD‐related spine dysfunction.

0N4R‐specific interactors also act at the presynapse: MARCKS controls synaptic fusion of RAB10‐positive vesicles (Xu et al. 2014). The Ras‐related GTPase RALA controls vesicle trafficking by interacting with the exocyst (Lalli 2009; Lalli and Hall 2005), a key complex for polarized exocytosis in eukaryotic cells (Hsu et al. 1998). In neurons, exocyst‐dependent exocytosis regulates neurite outgrowth, synaptic receptor transport, and neuronal polarity (Lalli 2009; Lalli and Hall 2005; Mehta et al. 2005; Sans et al. 2003; Vega and Hsu 2001). Two more 0N4R‐interactors, RAB11A and RAB11B, interact with multiple exocyst proteins and mediate vesicle tethering, as shown in yeast (Das and Guo 2011; Sultana and Novotny 2022). Of note, RAB11 has implications in AD pathogenesis as it controls the endosomal recycling of beta‐secretase (Li et al. 2012), and reduced RAB11 levels promote BACE‐mediated APP cleavage and accumulation of pathogenic amyloid‐β (Sultana and Novotny 2022; Udayar et al. 2013).

While it is known that Tau binds to presynaptic proteins and contributes to synapse dysfunction in AD (McInnes et al. 2018; Tracy et al. 2022; Zhou et al. 2017), we provide the first evidence that presynaptic interactions are isoform‐specific and that 0N4R‐Tau specifically binds signaling proteins modulating the exocyst‐dependent exocytosis and RAB11 protein function.

### 4.5 The Tau N‐Terminal Half Accumulates in Peroxisomes While Axonal Tau Specifically Binds the PP2A Activator HSP110

The identification of sorting‐specific Tau interactors is crucial to understand the Tau sorting process and to tackle pathological Tau missorting, an early pathological hallmark (Arendt et al. 2016; Guo et al. 2017; Tracy and Gan 2018).

When we compared the interactome of Tau isoforms (0N3R, 0N4R) and non‐sorting Nterm‐Tau, we found numerous Nterm‐specific binding partners associated with peroxisomes, the major organelle for metabolizing fatty acids and other lipids (Eckert and Erdmann 2003; Wanders and Waterham 2006). Interactions between Tau and peroxisomes were not described for both healthy and disease conditions (Kavanagh et al. 2022). When Tau undergoes proteolytic cleavage in disease, fragments similar to our constructs are generated (Guo et al. 2017), but they show mitochondrial (Corsetti et al. 2008; Corsetti et al. 2015) or untargeted localization (Ferreira and Bigio 2011; Park and Ferreira 2005). We found interactions with components of the major peroxisomal import complex (Platta and Erdmann 2007), but the mode of import remains unclear since all required recognition motifs are missing (Brocard and Hartig 2006; Lametschwandtner et al. 1998; Neuberger et al. 2003a, 2003b) and the construct was classified as ‘on‐targeting’ by two *in silico* prediction tools.

Out of the proteins that were specifically enriched for the Tau isoforms, the heat shock protein HSP110 is a well‐described interactor of axonal Tau (Kavanagh et al. 2022) and the major Tau phosphatase PPA2 (Arendt et al. 2016; Eroglu et al. 2010). Axonal PP2A exhibits manifold functions, as it is involved in cytoskeletal organization (Bhattacharjee et al. 2022) and promotes axon elongation, guidance, and branching (Lu et al. 2013; Ogura et al. 2010; Zhu et al. 2010). Downregulation of PP2A is associated with AD pathology, as it leads to Tau hyperphosphorylation, enhanced Aβ levels, increased neurodegeneration, and impaired spatial memory in rodents (Martin et al. 2013; Wang et al. 2015). Axonal Tau shows enhanced retrograde diffusion after PP2A loss‐induced hyperphosphorylation (Li et al. 2011). Our findings of somatic Tau accumulation upon treatment with okadaic acid corroborate the role of PP2A in axonal Tau retention.

Interestingly, depletion of HSP110 causes hyperphosphorylation of axonal Tau (Eroglu et al. 2010). Thus, HSP110 acts as a PP2A activator and could be critical for efficient axonal retention of Tau by promoting its MT binding affinity. Whether the binding of axonal Tau to HSP110 influences PP2A regulation, remains elusive.

## 5 Conclusion

Somatodendritic missorting of Tau is an early and crucial event in AD‐related Tau pathology, but knowledge about Tau‐intrinsic factors and Tau binding partners that mediate the axonal Tau sorting is sparse. Here, we provide evidence that the N‐terminal tail of Tau, its C‐terminal repeat domains, AD‐associated phosphorylation sites, and the general MT affinity of the protein play a minor role in axonal Tau enrichment in human mature iPSC‐derived neurons. In turn, we postulate that the PRR2 domain is a major regulator of axonal Tau sorting by a mechanism independent of AT8 phosphorylation. While the non‐sorting Nterm‐Tau accumulates inside peroxisomes, we identified the PP2A activator HSP110 as an axonally sorted Tau binding partner, implying a potential role of PP2A activity in isoform‐independent axonal Tau retention. In the isoform‐specific interactome, we found interaction with pathways that are involved in pre‐ and postsynaptic plasticity and associated with AD‐related synaptic dysfunction, such as the CDC42 pathway or RAB11 proteins. These findings indicate the differential role of Tau isoforms both in basic physiological function and Tau‐associated disease development.

## Author Contributions

Study design: M.B.S., H.Z. Experimental work: M.B.S., J.K., H.B., L.W., H.C, Methodological support: S.B., D.A. Data analysis and interpretation: M.B.S., H.Z. Manuscript writing: M.B.S., H.Z. Manuscript proofreading: S.B., J.K., L.W.

## Conflicts of Interest

The authors declare no conflicts of interest.

## Supporting information


**Figure S1:** acel70215‐sup‐0001‐FiguresS1‐S8.pdf.

**Table S1:** acel70215‐sup‐0002‐TableS1‐S11.xlsx.

## Data Availability

The data that support the findings of this study are openly available in ProteomeXchange at https://www.proteomexchange.org/, reference number PXD053880.
