# Supplementary figures and images for: Tau Axonal Sorting and Interaction With Synaptic Plasticity Modulators Is Domain‐ and Isoform‐Dependent in Human iPSC‐Derived Neurons
Source: Aging Cell. 2025 Sep 24;24(11):e70215. doi: 10.1111/acel.70215 (PMC12611310; doi:10.1111/acel.70215)

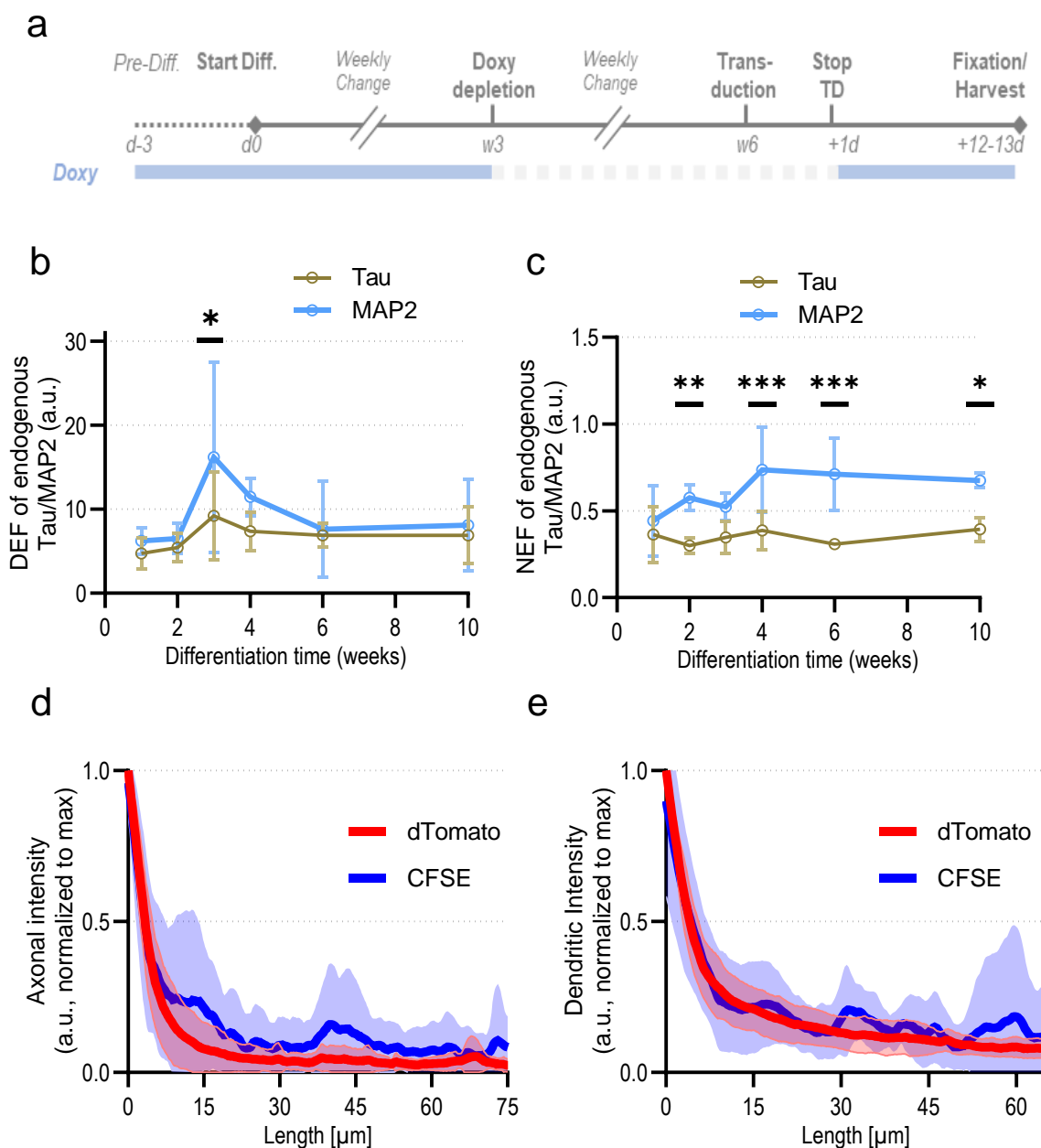

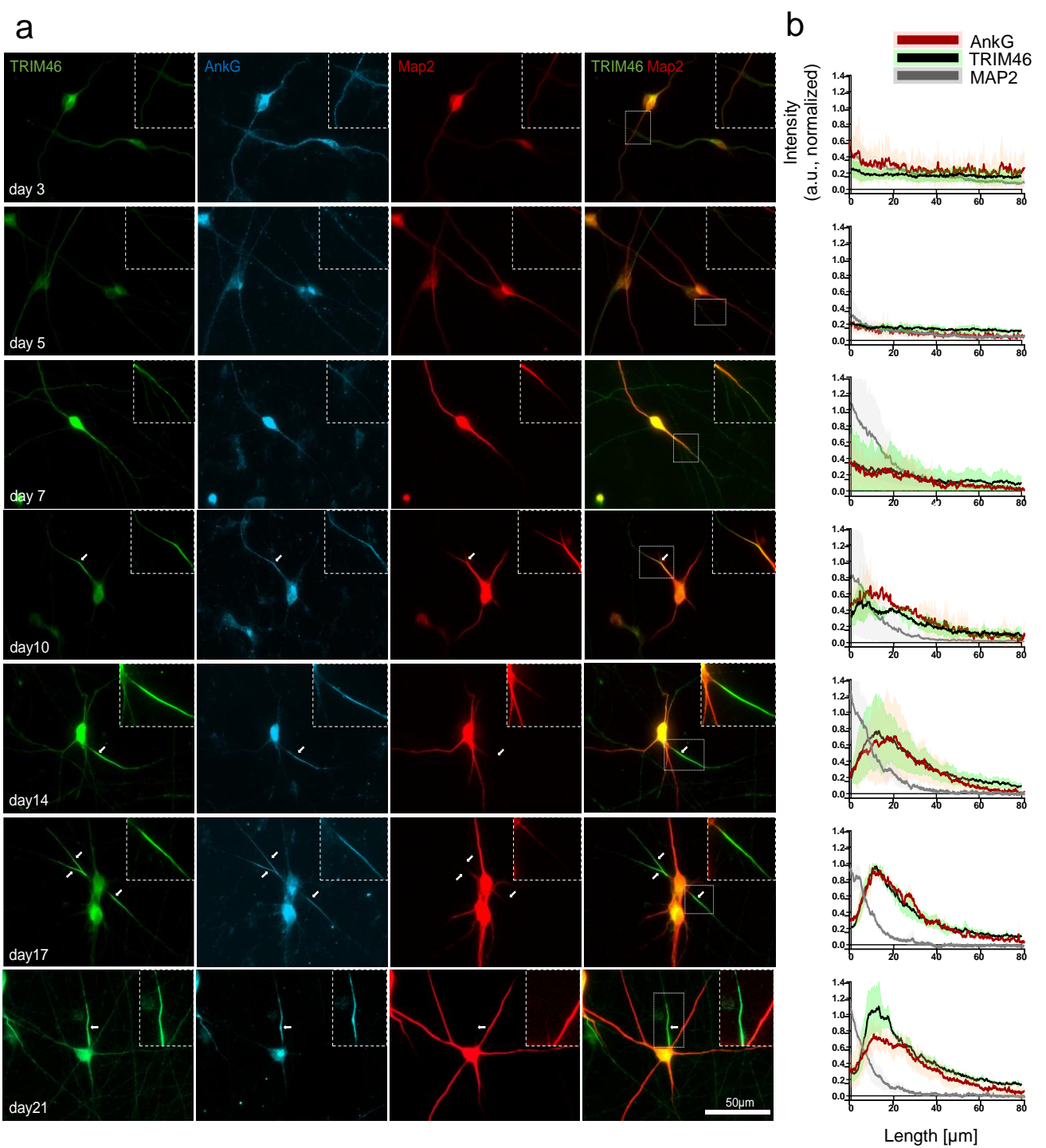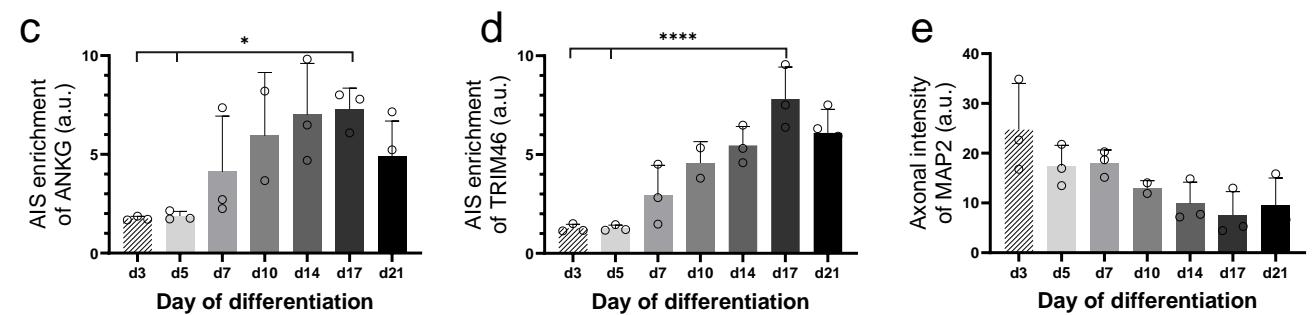

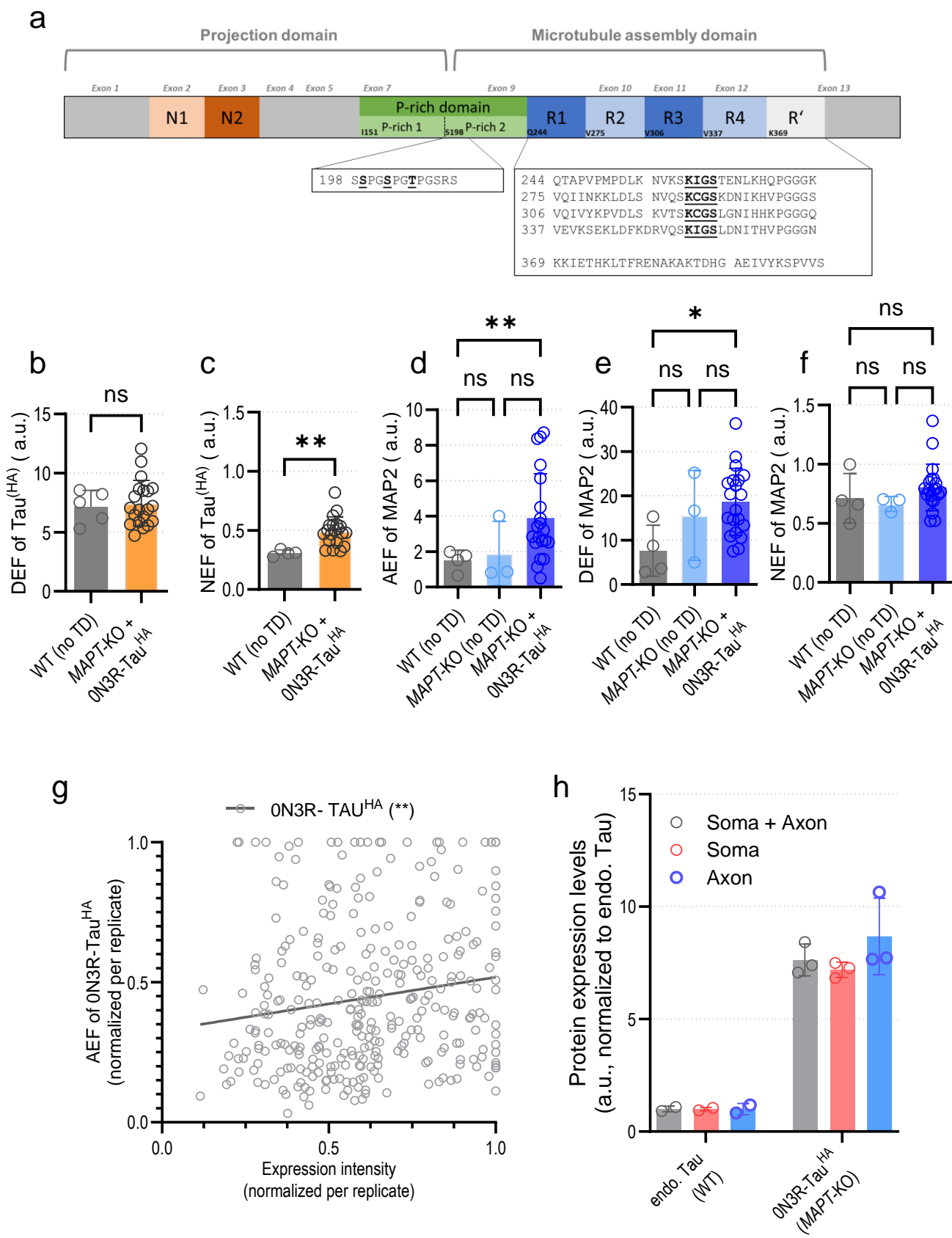

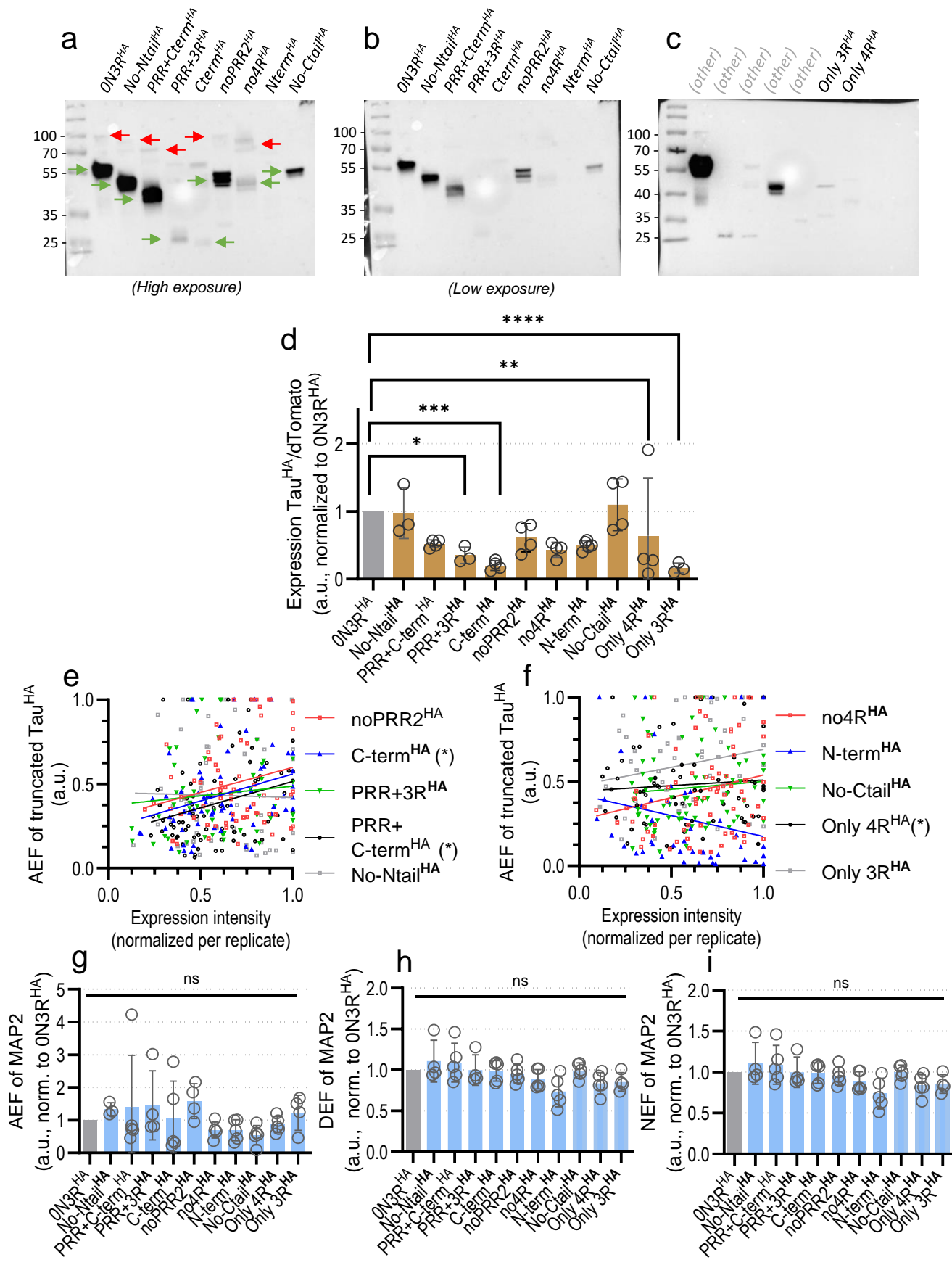

Suppl.Fig. 5

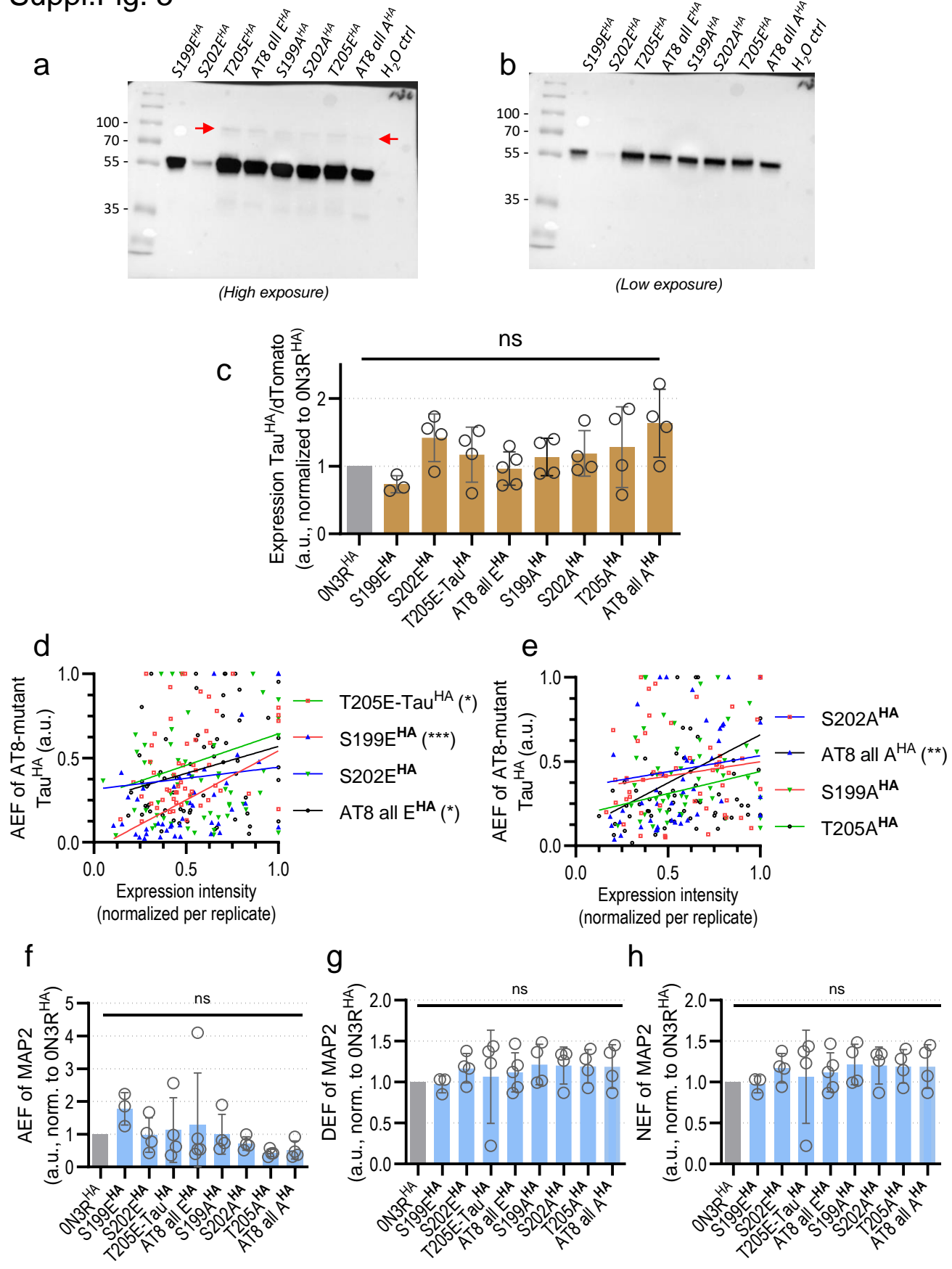

Suppl.Fig. 6

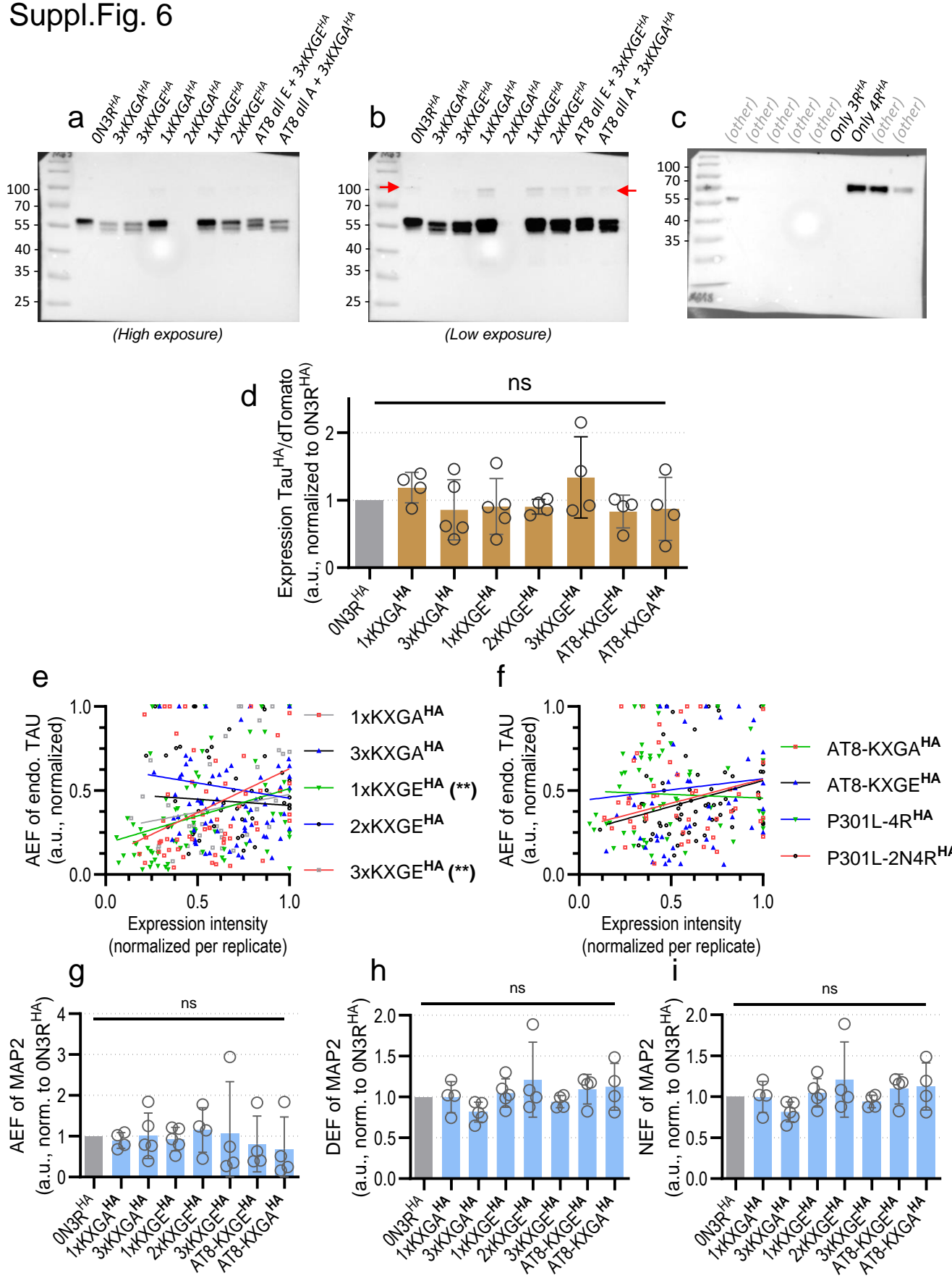

Suppl.Fig. 7

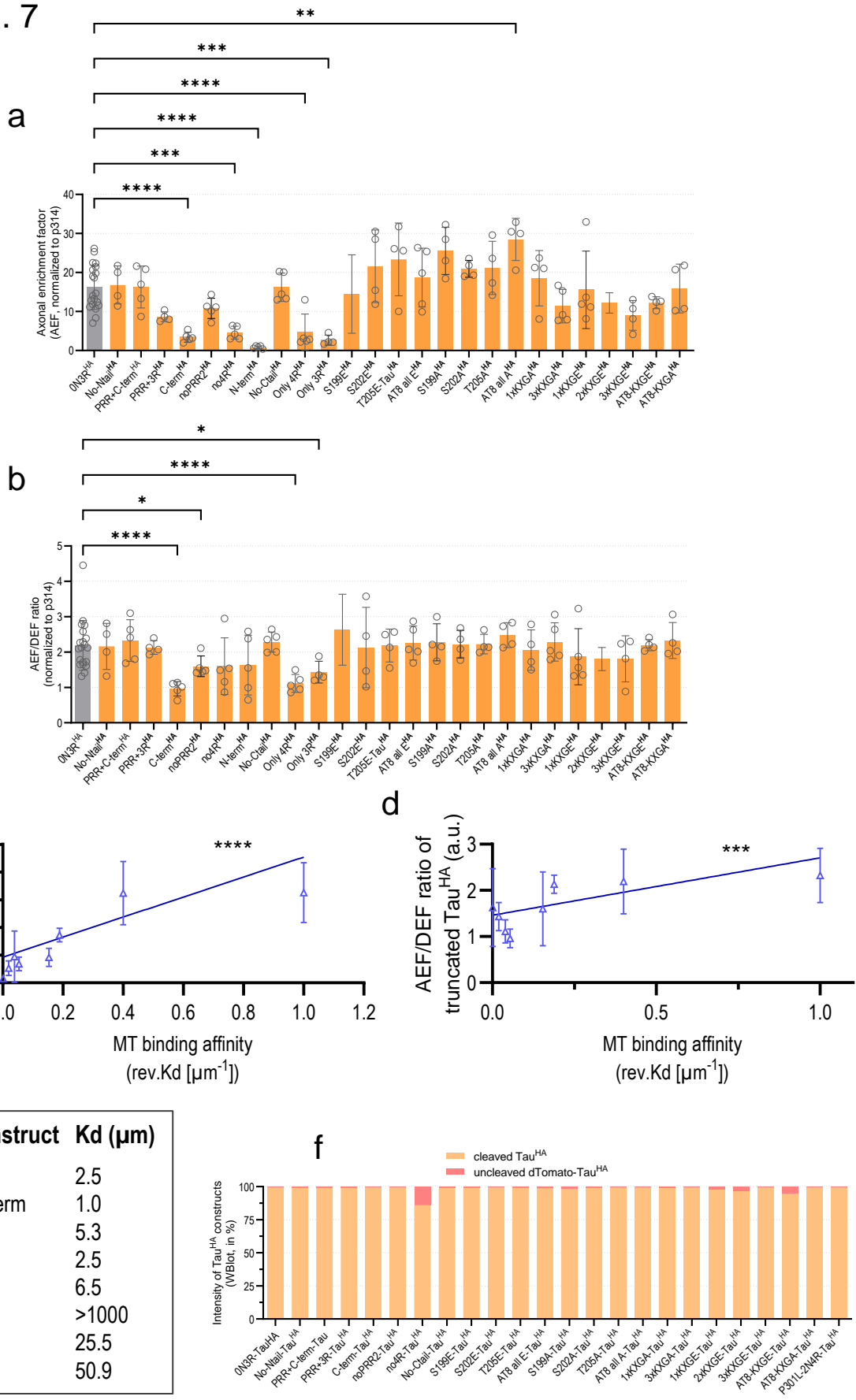

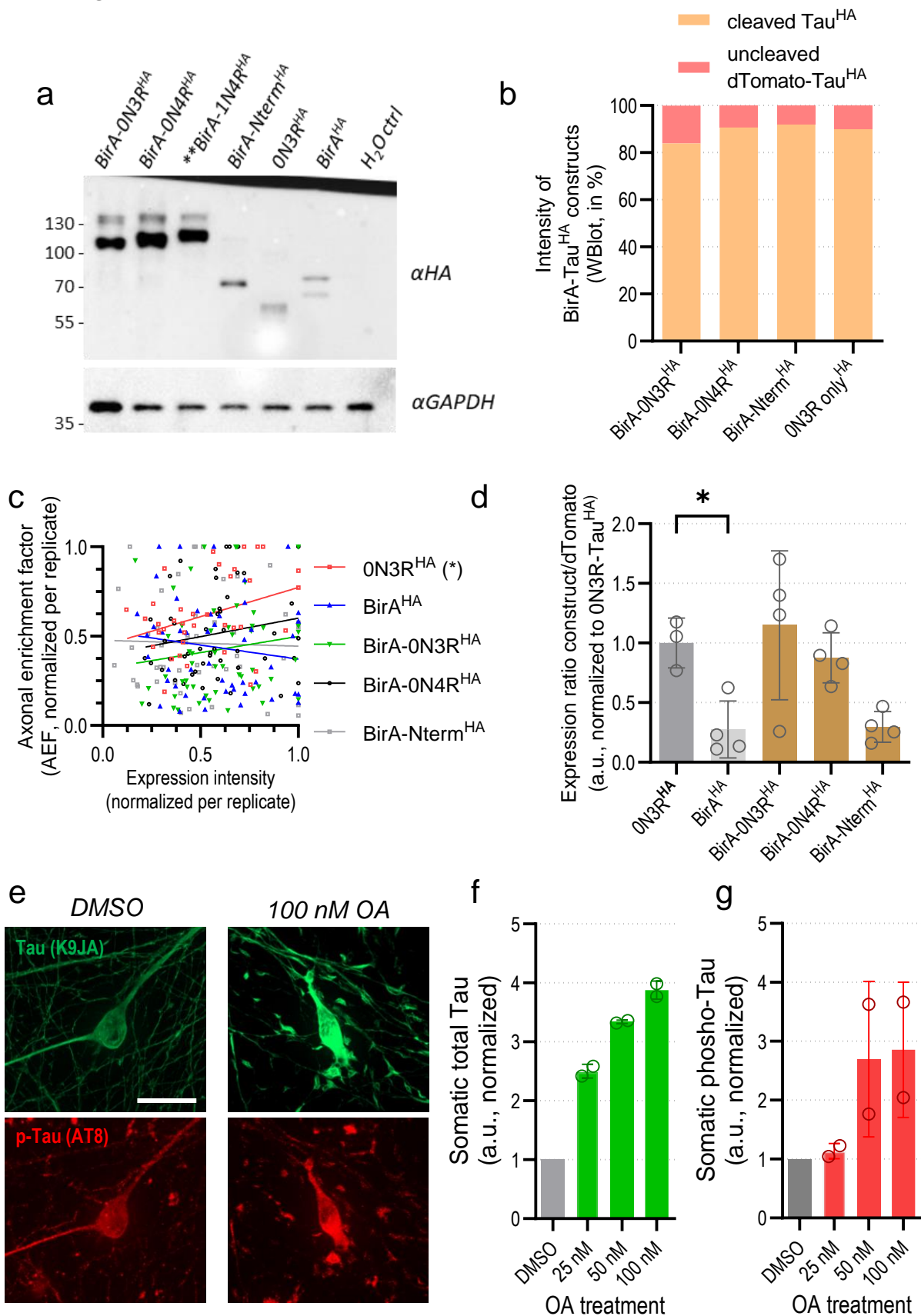

Supplement: Supplementary file 1 — Figure S1: acel70215‐sup‐0001‐FiguresS1‐S8.pdf. [file ACEL-24-e70215-s002.pdf]
